# Sustainable Use of Bioactive Compounds from *Solanum Tuberosum* and *Brassicaceae* Wastes and by-Products for Crop Protection—A Review

**DOI:** 10.3390/molecules26082174

**Published:** 2021-04-09

**Authors:** Daniela Pacifico, Chiara Lanzanova, Eleonora Pagnotta, Laura Bassolino, Anna Maria Mastrangelo, Daniela Marone, Roberto Matteo, Roberto Lo Scalzo, Carlotta Balconi

**Affiliations:** 1 CREA Council for Agricultural Research and Economics—Research Centre for Cereal and Industrial Crops, 00198 Rome, Italy; chiara.lanzanova@crea.gov.it (C.L.); eleonora.pagnotta@crea.gov.it (E.P.); laura.bassolino@crea.gov.it (L.B.); annamaria.mastrangelo@crea.gov.it (A.M.M.); daniela.marone@crea.gov.it (D.M.); carlotta.balconi@crea.gov.it (C.B.); roberto.matteo@crea.gov.it (R.M.); 2 CREA Council for Agricultural Research and Economics—Research Centre for Engineering and Agro-Food Processing, 00198 Rome, Italy; roberto.loscalzo@crea.gov.it

**Keywords:** sustainable food industry, waste residues, potato peel, brassica defatted meals, antifungal activity, mycotoxigenic fungi control, cereal protection, circular economy

## Abstract

Defatted seed meals of oleaginous Brassicaceae, such as *Eruca sativa*, and potato peel are excellent plant matrices to recover potentially useful biomolecules from industrial processes in a circular strategy perspective aiming at crop protection. These biomolecules, mainly glycoalkaloids and phenols for potato and glucosinolates for Brassicaceae, have been proven to be effective against microbes, fungi, nematodes, insects, and even parasitic plants. Their role in plant protection is overviewed, together with the molecular basis of their synthesis in plant, and the description of their mechanisms of action. Possible genetic and biotechnological strategies are presented to increase their content in plants. Genetic mapping and identification of closely linked molecular markers are useful to identify the loci/genes responsible for their accumulation and transfer them to elite cultivars in breeding programs. Biotechnological approaches can be used to modify their allelic sequence and enhance the accumulation of the bioactive compounds. How the global challenges, such as reducing agri-food waste and increasing sustainability and food safety, could be addressed through bioprotector applications are discussed here.

## 1. Introduction

Food loss and waste are a complex problem involving various drivers along the food supply chain. The worldwide intensification of agro-industrial processing and the current linear food economy based on converting natural resources into waste lasted so long, creating large quantities of residues, and their management poses economical, hygienic, and environmental problems [1]. The future state of global natural resources, especially in the frame of the current climatic change, forces the world towards a turnaround in order to achieve significant outcomes in waste reduction, though keeping high yield production levels and improving accessibility and quality of the resources. Among the possible solutions, international organizations implement the idea of sustainable development, which satisfies the requirements of the circular economy in the view of a new agro-industrial system (UN 2030 Agenda for Sustainable Development, the UN Convention on Biodiversity Strategic Plan for 2020, the EU Green Deal “Farm to Fork strategy” for a fair, healthy, and environmentally friendly food system and the upcoming Horizon Europe 2021-2027 Pillar II-Cluster “Food, bioeconomy, natural resources, agriculture and environment”).

The challenge for the circular economy to implement the sustainability of a modern agricultural management is facing the increase in human population, the request for high crop yield, food production, and safety. To achieve these goals, the main issue to be solved is to guarantee a sustainable and circular way to control crop pests. The most common tool used for crop protection consists of synthetic pesticides, which offer a practical and rapid solution to plant pathogen disease outbreaks. However, numerous problems arise from the wide-scale use of chemical pesticides: Their negative impact on environment [2], several fruit and vegetable industrial processing residues [3], and the development of fungal resistance to their active substances [4]. Therefore, the use of chemicals has long been viewed as a strategic management to control crop diseases, and nowadays, EU farmers have to manage plant protection without many pesticides because of the obligation of their gradual dismissal [5]. The sprout inhibitor Chlorpropham used to protect stored potatoes has been recently banned (January 2020), as was Chlorothalonil, a fungicide for wheat and barley protection, banned from May 2020, and Mancozeb, approved in the EU in 2006 for very wide use, especially for controlling potato blight, and under increasing scrutiny from the EU Commission and from member states. With the aim to highlight alternative tools to chemical products, researchers started to be strongly focused on innovative strategies such as identifying plant molecules useful to develop natural biocides [6].

Botanical extracts can be effective in controlling a wide range of plant pathogens providing an innovative and sustainable solution to be applied in organic and conventional agriculture. Studies on extracts from garlic, carnation, cinnamon, and thyme showed, for example, their effectiveness on *Fusarium solani* and *Rizochotonia solani* in faba beans [7]. Similarly, lupine seeds were successfully treated with extracts of *Nerium oleander*, *Eugenia jambolana,* and *Citrullus colocynthis* [8]. Clove, garden quinine, Brazilian pepper, anthi mandhaari, black cumin, white cedar, neem, and rice extracts were used in the control of leaf rust in wheat [9,10]. It is unclear if these products can improve plant vigor and response to other fungal pathogens, especially under organic farming conditions, in which plants are more susceptible to the pathogen-induced damage. These extracts could act by enhancing the host defense mechanisms [11], either by increasing the activity of peroxidase, the accumulation of phenolic compounds [12], or through inhibition of some antioxidant enzymes and catalases, thereby leading to the production of elevated amounts of H_2_O_2_ [13].

In view of the increasing interest in the concept of circular economy, using molecules recovered from other productive activities, rooted in the territory with no further input of new matter, which will reduce the impact of pathogens, pests, biotic stresses on cereal crop quality and yield, is of strategic importance to guarantee food for new generations with respect to the future state of global natural resources. Minimizing the ‘yield gap’ and increasing yield stability under different stress conditions is the worldwide trend being emphasized. However, despite the increasing interest around the concept of circular economy, there are currently few systematic studies on extracts from wastes of *Solanaceae* and *Brassicace*ae that can explain their effects on plant pathogens due to a defined concentration of bioactive molecules [14].

Potato is the fourth major food crop in the world, with a production around 388 million tons [15], of which <50% is consumed fresh [14]. The large consumption of processed potato products (French fries, potato crisp, and frosted potatoes) causes a huge quantity of residues every year as Potato Peel Waste (PPW), a by-product of the large-scale peeling from manufacturing industries [16]. This amount accounts for 15 to 40% of the initial product’s mass depending on the peeling methods [17,18] and ranges between 70,000 and 140,000 tons worldwide [19], posing a pressing challenge of how to use it [16] in order to avoid considerable environmental and economic concerns, such as pollution derived from organic waste decomposition. It has been a long time that the whole tuber can be converted into energy, as a source for biogas, biofuel, and biomanure [20], but this destination of use does not consider the high valuable utilization of peel. The presence of active compounds such as phenols and glycoalkaloids should favor the area of re-use or recovery, i.e., of green extracts suitable as nutraceutical and drugs [21] and as additives for improving the shelf life of fresh-cut fruits [22].

Concerning *Brassicaceae*, a green biorefinery approach was successfully applied in recent years in particular to oleaginous *Brassicaceae* with the aim to create novel agriculture inputs such as fertilizers, amendments, biostimulants, and biopesticides for the control of pest and pathogens both at the open-field level and in the post-harvest phase [23]. Vegetable oils, in particular those from *Brassica* oilseeds, are well known for their potential applications in green chemistry, including their use as hydraulic fluids, biolubricants, and cosmetics [24]. A fair biorefinery approach, devoted to the production of oil as the main product, should be closely related, in the frame of circular economy, to the exploitation of one or more co-products with the aim of minimizing or avoiding waste production [25]. Biomolecules produced from *Brassica* oilseed co-products, which can be applied in industry and as sustainable tools in agriculture, represent an urgent option considering the health and environment problems caused by conventional chemical application. The perspective of fractioning the *Brassica* co-products permits the separation of high added molecules, allowing the preparation of a pool of bio-based materials useful for several applications, which is a fundamental approach for innovative biorefineries [23].

This review aims to provide a comprehensive perspective of the scientific literature focused on the biological role of secondary plant metabolites derived from potato and *Brassicaceae* wastes such as alkaloids, glycoalkaloids, terpenoids, organic acids, glucosinolates (GSLs), and their hydrolysis products. These biocompounds are promising sources of plant-protecting tools and could be used as bio-weapons against pests, for example reducing the attractiveness of plants to different insects or reducing fungal growth development [26,27,28,29].

## 2. Biocompounds in Potato Peel

Potato germplasm is characterized by a huge variability in composition and concentration of secondary metabolites that play a role in increasing plant ability to cope with environmental challenges, due to their reported biocide activity on insects, bacteria, and fungi [27,30,31]. Their distribution within the tuber is not uniform: Most of them are concentrated in the peel, made of periderm tissue, whose cell layers contain corky cell walls, which confer protection from phytopathogens, especially during tuber growth and storage. Thus, considering that potato peel is constantly exposed to biotic stresses, it is not surprising that it is a precious source of bioactive compounds, mainly phenolics and alkaloids, which have an enormous potential to deliver new bioprotectors.

***Potato Glycoalkaloids***

**Figure 1:**
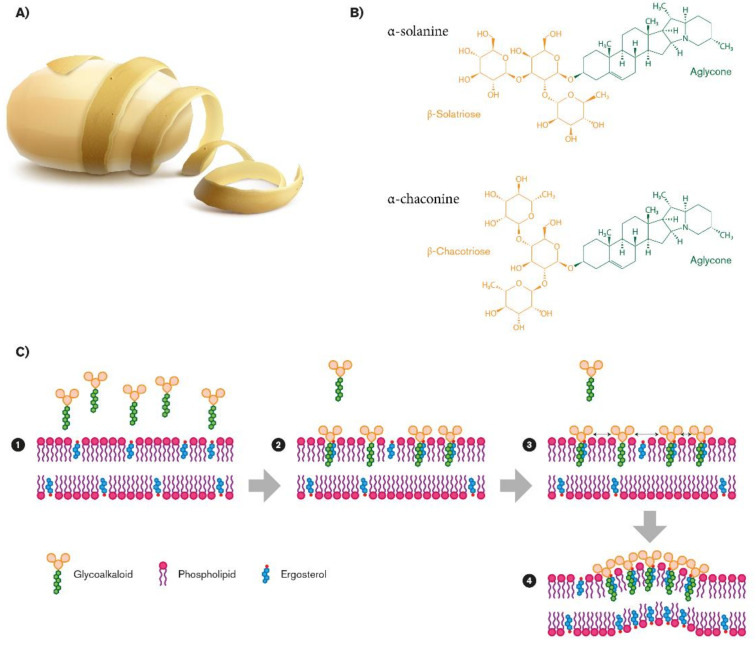
Biovalorization of glycoalkaloids α-chaconine and α-solanine from potato peel waste (**A**). Their chemical structures (**B**), and their role in fungal membrane disruption (**C**).

Steroidal glycoalkaloids (SGAs), a class of nitrogen-containing steroid glyosides, are the most important alkaloids in the *Solanaceae* family [32]. In potato germoplasm, there are more than 80 different molecules [33,34,35], but in cultivated potato, α-chaconine (β-d-Glucopyranoside, (3β)-solanid-5-en-3-yl O-6-deoxy-α-l-mannopyranosyl-(1-2)-O- (6-deoxy-α-l-mannopyranosyl-(1-4)), and α-solanine (solanid-5-en-3β-yl α-l-rhamnopyranosyl -(1→2)-[β-d-glucopyranosyl- (1→3)]-β-d-galactopyranoside) account for up to 95% of the total tuber SGAs [36,37]. SGA structures share a common aglycone, a six-ring steroid skeleton (solanidine), to which a branched triose is attached. The triose is a sugar moiety attached to the 3-position of the first ring and a nitrogen atom in the sixth ring end of the molecule: In the case of α-chaconine it consists of β-chacotriose (bis-α-l-rhamnopyranosyl-β-d-glucopyranose), while for α-solanine, it is a β-solatriose (α-l-rhamnopyranosyl-β-d-glucopyranosyl-β-galactopyranose (Figure 1B) [36]. The SGA biosinthetic pathway has not yet been completely determined, even though it putatively derives from isopropanoid pathway. Cholesterol has been identified as metabolic precursor: It is cyclized into solanidine, a steroidal alkaloid (SA) that is subsequently glycosylated to α-solanine and α-chaconine [38]. A similar SGA, tomatine, has been found in tomato and eggplants, with small but significant structural differences. It has a branched chain composed of lycotetraose, a tetrasaccharide constituted by two glucose units, xylose and galactose (22S,25S)-5α-spirosolan-3β-yl-β-d-glucopyranosyl-(1→2)-[β-d-xylopyranosyl-(1→3)]-β-d-glucopyranosyl-(1→4)-β-d-galactopyranoside [36].

The distribution of SGAs in plant is not uniform, including leaves, tubers, roots, and sprouts, especially in green parts, flowers, and fruits [36]; their accumulation is affected by many different factors, including developmental and environmental conditions such as high temperature [39], light exposure [40], light quality [41], and wounding [42], but it also depends on genotype [43]. SGA content in the peel was often reported to be high, ranging between 0.83 and 352.6 mg/100 gr DW (dry weight) in eight potato cultivars [44] and resulting in 50 mg/100 gr DW in *cv* Bionica [45]. Two interesting studies indicated that this variability could be correlated with flesh color: White-flesh potatoes showed higher content of SGAs in peel than blue-flesh, red-flesh, and yellow-flesh potatoes [46]. White-flesh potatoes can reach up to 3526 mg Kg^−1^, blue-flesh potatoes 245 mg Kg^−1^, red-flesh potatoes 1264 mg Kg^−1^, and yellow-flesh potatoes 425 mg Kg^−1^ FW (fresh weight) [47].

SGAs are considered toxic to humans: Daily oral doses from 3 to 6 mg GAs/kg body weight can even be lethal [48]. The widely accepted safety limit in tubers is 20 mg/100 g FW, even though a maximum level has not been officially established at the EU level [43]. A recent opinion from the German Federal Institute for Risk Assessment (BfR) suggests lowering the safety limit in potatoes to less than 10 mg/100 g FW [49]. However, most edible mature tubers contain low amounts of SGAs in their flesh [50], as a consequence of their selective reduction by breeding due to potato domestication for human consumption. Commonly commercialized potatoes rarely exceed the recommended SGAs level, with some exceptions such as *cv.* Lenape and *cv*. Bonum that were consequently withdrawn from the US and Swedish markets [51,52].

***Potato Phenols***

Thousands of phenolic compounds have been isolated in the plant kingdom with a role of either oxidative stress protectors or as pest control agents, and have been classified in several subgroups based on their structure: Phenolic acids, flavonoids, tannins, coumarins, lignans, quinones, stilbens, and curcuminoids. Their structure contains hydroxylated aromatic rings, with the hydroxy group being attached directly to the phenyl, substituted phenyl, or different aryl group. Phenolic compounds are synthesized via the shikimic acid and phenylpropanoid pathways. Potato peel is a well-established source of phenolic acids and flavonoids: Their content varies, respectively, among 1.02–2.92 g/100 g and 0.51–0.96 g/100 g DW [21] and significantly decreases toward the skin close sections (cortex) and flesh, as confirmed either by direct measurement, such as HPLC analysis [45], and indirect measurement, such as the radical scavenging activity [53]. The most abundant phenolic compound is chlorogenic acid, CGA (5-O-caffeoylquinic acid; 5-CQA), together with its isomers (3,4-diCQA, 3,5-diCQA, and 4,5-diCQA) [36]. CGA was also truly called 3-caffeoylquinic acid before 1976, when, according to new IUPAC rules, the CA structure changed in 5-caffeoylquinic acid and the 3-caffeoylquinic acid isomer was referred to as neochlorogenic acid [54]. CGA resulted in nearly 2115 µg g^−1^ DW in skin, decreasing in adjacent cortex to 276 µg g^−1^ DW (*cv*. Bionica) [45]. Conversely, hydroxycinnamic and hydroxybenzoic acids are present only in trace, with the exception of caffeic acid ((2*E*)-3-(3,4-dihydroxyphenyl) prop-2-enoic acid; CA). CA and its derivatives, and catechin (C_6_-C_3_-C_6_), belonging, respectively, to the class of hydroxycinnamics (C_6_-C_3_) and flavonoids, specifically flavan-3-ols, resulting in the lowest concentration in the tuber.

CA possesses relevant antioxidant activity both in vitro and in vivo, higher than that observed for CGA [55] and it can be accumulated in plants, mainly in conjugated forms due to esterification by quinic acid (1*S*,3*R*,4*S*,5*R*)-1,3,4,5-tetrahydroxycyclohexane -1-carboxylic acid), in two configurational isomers, such as *trans* or *cis*. In biological systems, the most relevant form is the *trans* isomer, because of its best stability at subacid pH in the plant microenvironment [56], even though its photoexcitation for the absorption in the UVA region (400-315 nm) can lead from *trans* to *cis* structure [57]. Its ortho-diphenolic moiety confers important antioxidant features to CA: Lowering the OH bond dissociation enthalpy, thereby increasing the rate of H-atom transfer to peroxyl [58]. Moreover, the *ortho*-diphenolic system has a relatively higher oxidative reaction rate, with the negative secondary effect of the tuber turning brown when cut or damaged, with the essential contribution of molecular O_2_ and of specific enzymatic activities. The molecular mechanism of the browning phenotype is not yet completely elucidated [59].

Catechin (2R,3S)-2-(3,4-dihydroxyphenyl)-3,4-dihydro-2H-chromene-3,5,7-triol) is an ortho-diphenolic belonging to the flavan-3-ol family. Differently from other phenol compounds, catechin is found in free form. Peculiarly, it can be condensed in oligomers or in higher molecular weight polymers, commonly named “condensed tannic compounds”, present in black tea and red wine [60]. Its chemical structure confers interesting antioxidant properties, higher than those of other common biological antioxidants, such as glutathione and ascorbate [61]. Although its level in peel is lower compared to CA, catechin might be investigated as a potential bioprotector due to its antimicrobial and antifungal activities [62].

### 2.1. Potato Eco-Friendly Plant Bioprotector Activity Against Biotic Stresses

#### 2.1.1. SGA Activity Against Phytophages

Chowansky reported an exhaustive study on SGA activity on insects, such as Heliothis virescens, Manduca sexta, Spodoptera frugiperda, Podisus maculiventris, Schizaphis graminum, Leptinotersa decemlineata, Ceratitis capitata, Empoasca fubae, Mizus persicae, Galleria mallonella, Tribolium custeneum, and Zophobas atratus [27]. The hypothesis about the mechanisms by which they act involve molecular, cellular, and organismal levels. As are most pesticides, SGAs are also inhibitors of the acetylcholinesterase and butyrylcholinesterase enzymes, which catalyze the hydrolysis of acetylcholine at the synapse in the nervous system [63]. These features are the main issues determining that they should not exceed limit level in human consumption and, at the same time, are the reasons why many studies on their biocide activity were performed. Additionally, the bitter/burning taste that SGAs confer lead to insect-feeding deterrence: The pre-ingestive antifeedant effects could be due to the taste sensations they impart, especially related to α-chaconine, while their putative synergism in antifeedant activity has not been shown yet [64,65]. Among the large number of studies on SGAs activity, most of them focused on the correlation between genotype-resistance and SGA content in vivo and on in vitro dose–response testing of pure metabolites against pests. The resistance of five potato genotypes to Phhorimaea operculella (Lepidoptera: Gelechiidae), the causative agent of potato tuber moth (PTM), was correlated to peel SGAs and peel phenolic compound contents, suggesting a putative key role of α-chaconine and CA in potato PTM resistance [45]. Data on sublethal or lethal toxicity of pure metabolites from a dose–response test are also available. On the whole, the activity of α-chaconine was frequently higher than α-solanine one by a factor of 3–10 times [64,66,67], with only a few exceptions [68], but their synergistic effects remain questionable, and the dose effect of pure SGAs solutions compared to their mixtures have been scarcely studied. Smith exhaustively discussed their activity on snail *Helix aspersa* L. [64] already known for its high susceptibility to SGAs [69]. In pure form, both singly α-solanine and singly α-chaconine showed an increasing feeding deterrence at higher concentrations. A combination of both synergistically increased the feeding inhibition. Additionally, in order to understand if peel could contain other substances that may interfere with SGAs, the authors compared the activity of the raw potato peel extracts with the equivalent concentration of SGAs solutions in mixtures. The peel extracts of cv. Majestic and cv. Sharpe’s Express did not show significantly different inhibition activity with respect to the equivalent SGA mixture. On the other hand, peel extracts of cv. Homeguard inhibited feeding more effectively than solutions of SGAs suggesting their presence in the peel of additional inhibitory biocompounds. The most interesting aspect is that cv Homeguard also showed lower SGAs content in the peel among the three evaluated genotypes. In 2011, Nenaah carried out a similar study on adults of the red flour beetle Tribolium castaneum Herbst and on the rice weevil Sitophilus oryzae L. [70]. The SGA activity increased in a dose-depending manner, and once more, raw peel extract resulted in being more toxic than pure molecules. Thus, a lack of correlation between the bioactivity of raw peel extract and the dose equivalence of the most active pure compounds, determined through the peel profiling composition, was observed. Antagonistic, additive, or synergistic interaction against the pest could be an explanation, such as concurrent or competitive binding of two or more compounds to the receptor site of the pest.

Similarly, Friedman [68] showed a high SGA activity in a dose–response test against human and animal pathogenic *Trichomonads* strains. Raw potato peel extracts have different antiprotozoal inhibitory effects depending on both genotype and strain, but, as observed in inhibition of complex organisms [64], peel of the *cv*. Russet, despite the lowest content of SGAs, showed the highest inhibitory effect, suggesting the presence of additional compounds able to influence SGA bioactivity modulating their final effect. Therefore, the various substances present in extracts may reciprocally increase [64] or decrease the toxic effects of SGAs [68].

Based on the synergistic effect of the two predominant SGAs, Gee and co-workers suggested that SGA activity against pests is more likely mediated through effects on the membranes of chemoreceptor cells rather than via signal transduction by neurotransmitter enzyme [71]. Indeed, SGAs are known to alter membrane potential/trans membrane ion transport and to synergize in this action. In vitro studies of SGAs indicate that they may have cytotoxic effects that often compromise cell membrane integrity, through the disruption of phosphatidylcholine/cholesterol liposomes [36,72], causing negative effect on intestinal permeability and altered metabolism [36,73]. This mechanism is also confirmed in frog embryo cells [74], rabbit erythrocytes, beet cells, and protoplast of *Penicillium notatum* [75], where α-chaconine has been observed as the most active compound.

#### 2.1.2. SGA Antifungal Activity

SGAs are inhibitory to a wide range of fungi. Fewel and Roddick have reported in vitro activity against four different fungi, not all potato specific (*Alternaria brassicicola, Phoma medicaginis Ascobolus crenulatus,* and *Rhizoctonia solani*) [66], and afterward, Cipollini and Levey have been proven against additional 10 different strains [63]. A synergism between α-chaconine and α-solanine in inhibiting the percentage of radial growth of *A. brassicicola* and *P. medicaginis* has been observed. Indeed, a significantly higher activity of inhibition of them was reported, upon coadministration of mixed compounds rather than pure ones [66]. The ratios producing their maximal synergism and maximal inhibition are of the order of those naturally occurring in *Solanum* species; however, an amount of them as little as 10-20% in a mixture in vitro was sufficient to activate an important synergism. Similarly, SGAs from tomato (solasonine and solamargine) showed the capacity to reduce the spore germination of *A. brassicola* and *P. medicaginis* either alone or in combination 1:1 [67]. Additionally, SGAs from potato were not responsible for the resistance to the oomycete *Phytoftora infestans*, the causative agent of the late blight disease responsible for major damage to potato crops [76]. *P. infestans* was also tested with SGAs from tomato and aubergine and the most interesting finding was that the last one appeared to exercise a greater toxic effect on the oomycete than compounds from potato, implying some level of host adaptation [77].

#### 2.1.3. Interaction Between SGAs and Fungal Membrane

A possible explanation of the great variation in SGA resistance among the fungi species is the composition of the sterol patterns present in the fungal membrane, closely related to the fungi taxonomic classification [78]. The SGA biological activity depends on their membrane disruption by which they compromise the structural and functional integrity of cells and tissues of the organisms [75]. Indeed, SGAs consist of an aglycone (solanidine) with a carbohydrate side chain thought to be important for the interaction with sterols of the fungal lipidic bilayer (Figure 1B). This property has been confirmed by solasonine and solamargine, using synthetic membrane vesicles, liposomes [79], and protoplasts [75]. The leakage of SGAs with membrane sterols suggests that the sterol pattern of fungal membranes influences SGAs activity on fungi. In liposomes, SGAs interact more efficiently with membrane sterols with planar ring structures and a three b-OH: β-sitosterol and fucosterol made the bilayer much more susceptible to SGAs than cholesterol and ergosterol, present in fungal membrane [80,81]. Recently, Sánchez Maldonado confirmed the membrane-disruptive effect of glycoalkaloids: α-chaconine, but not α-solanine, disrupted phosphatidylcholine/cholesterol liposomes, and confirmed that the fungal resistance to α-chaconine is due to the sterol pattern, particularly related to the relative presence of some specific unsaponifiable lipids [82]. α-chaconine resulted in being invariably more damaging than α-solanine, and the synergism in sterol binding significantly enhances the membrane-disruptive activity of SGA mixtures, even though this effect has only been demonstrated in vitro [82].

The proposed mechanism of activity involves insertion of the aglycone in the bilayer in an aglycone/sterol ratio of 1:1 (step1 and step 2), followed by sugar–sugar interactions between the sugar moieties of glycoalkaloids that start the formation of an irreversible complex (step 3; Figure 1C). As a result, a rigid sterol-glycoalkaloid matrix is formed (step 4), which disturbs membrane function and causes lysis of the cell (Figure 1C) [80]. This hypothesis is also supported by the loss of activity after cleaving monosaccharides from the glycosidic moiety of glycoalkaloids in liposomes containing sterols [80].

The sterol profile of fungi, a chemotaxonomic tool [78,83,84], seems to relate to their resistance to SGAs due to their effective interface with fungal membrane. The same mechanism could be also the explanation as to why a pH dependency of SGAs on fungal growth inhibition was observed: their effect was generally lower at pH 6 than at pH 7. At lower pH, this behavior could be hampered by a decreased alkaloid solubility in a lipophilic environment, due to an increased protonation of the steroidal N-moiety. Indeed, it has been shown that the successful invasion of tomato fruits by fungal pathogens was related to the ability of the fungus to decrease the pH and hence the activity of tomatine [66,85]. This fact could be the consequence of fungal detoxification capacity through pH change [86]. However, at pH 6, a valuable SGA synergism was observed in all fungi tested whose magnitude decreased at pH 7. A possible explanation is the masking effect due to the higher activity of individual glycoalkaloids at higher pH.

Moreover, fungal resistance to SGAs activity can also depend on hydrolytic enzyme secretion [87]. Extracts derived from virulent fungi contain enzymes with SGA degrading activity. In that way, some phytopathogenic fungi can overcome SGA toxicity by their enzymatic deglycosylation: The removal of the trisaccharide from SGAs would lead to no toxic steroidal alkaloid (SA) solanidine. Only a few exceptions are reported. In a recent study, solanidine resulted in being more active than glycosylated forms to inhibit the growth of *P. infestans* [77]. Very recently, a bacterial gene cluster (isolated from *Arthrobacter* sp. S41) involved in the complete deglycosylation of SGAs has been characterized, as suitable for a potential application in the bioconversion of feed proteins to food ones, useful for human nutrition [88].

#### 2.1.4. Role of Phenols in Plant Protection

The role of phenols in defense against predators and diseases in plants seems to be well documented. For example, in potato skin, CGA and CA contents are strongly related to PTM larval mortality [45]. However, dose–response testing of pure phenols from potato (CA, CGA, and quercetin) showed only mild inhibition activity against *Thricomonas* [68]. Moreover, CA alone showed only weak antifungal activity when compared to other phenolics and alkyl esters tested, showing the lowest minimum inhibitory concentration but also the highest protection factor [89,90]. It would be interesting to study, in depth, whether the phenols characterized by limited inhibitory effects, when tested alone, could instead show the ability to synergize with SGAs. Indeed, the effect of SGAs could be strongly enhanced by phenols. Accordingly, CA increases the α-chaconine activity against fungi 1000 times, decreasing its minimum inhibitory concentration and the physiological mechanism causing a synergistic effect with SGAs; a change in membrane fluidity could be the reason [81]. The possibility of an added effect given by catechins and their derivatives, also well known as modifiers of membrane fluidity, could be a useful tool to enhance this biological process [91], but up to now, no studies on it have been reported. In Table 1, the literature about SGA and phenolic activities is summarized.

### 2.2. Recovery of Eco-Friendly Bioprotectors from PPW

In the framework of an eco-friendly, integrated, and circular agro-economy, the demand for reducing waste through the re-use of the residues from agro-industry, during the last years, has been relevant. For this reason, the release of the environmental pressure from potato peel, considered as one of the most important agro-wastes [16], and the high PP surplus value due to rich biocompound content [92], confer to PPW an enormous potential to deliver new products for crop protection [21]. Highlighting PPW recovery potential benefits and considering the connected limits will have a huge impact on production-integrated systems through the development of new highly sustainable products and tools suitable for low-impact agriculture and organic farming systems.

To date, information concerning a bio-formulate based on SGAs and phenols to be used in organic defense or in Integrated Pest Management (IPM) is not available. On a dry weight basis, PP represent 5 to 9% of the whole potato and often contains 50% or more of the total SGAs in the whole tuber [44]. The fact that environmental agro-technological factors, together with the genotype, may greatly influence chemical composition is well known; for example, SGAs and phenols are higher in peels from organic tubers, with the only exception being the red genotype [93]. Thus, the determination of SGAs and phenolic content in the peel of the most-used potato-processing cultivars grown in different agro-technical regimes (conventional and organic tubers) would provide useful information to manage the levels of secondary metabolites. In addition, post-harvest technology of potato storage has been historically aimed at lowering the levels of SGAs in edible tuber as soon as possible, but factors such as light, temperature, humidity, wounding, and processing conditions may elevate SGAs content to toxic level [94].

A crucial and limiting issue is the optimization of the industrial multi-step process in the frame of a green strategy necessary for sustainable PPW exploitation [14]. The first critical point to be addressed is how to dry the starting fresh sample to obtain a stable and extractable matrix. A non-conventional, effective, and cheap approach could be solar drying [95]. However, it can induce a variable retention of phytochemicals in comparison with conventional techniques. Indeed, a forced-air oven, moved by electric power, still leads to a well-dried product in a short amount of time [96], even though it has high energy consumption. The following step, the extraction of bio-compounds from PPW, is the most critical, to a great extent depending on the choice of solvent to be used in the process. Actually, the best procedure for phytochemical extraction employs protocols including the use of alcohols, usually ethanol, and is characterized by long extraction times, a large quantity of solvent, and high temperature. The utilization of supercritical systems of fluids, although very expensive and not eco-friendly, resulted in being very effective. The “green” extraction alternative protocol should employ the use of water, but often this solvent is not effective, especially because of the low yields [97]. Ultrasound-assisted extraction (UAE) is a promising and innovative technique, well described by Bankeblia [14] and Hossain [98]. Additionally, the characterization and quantification of active principles in recovered by-products is carried out at the lab-scale by using complex chromatographic methods, such as HPLC (high-performance liquid chromatography) and HPTLC (high-performance thin-layer chromatography). For this reason, a future perspective to improve the sustainable bio-compound extraction procedure from PPW could be based on the development of user-friendly and non-destructive monitoring methods, already applied in the internal quality evaluation process of some fruits [99], by means of devices easily used by non-specialized personnel. To date, these non-destructive approaches are not validated and do not fulfil economic and environmental criteria, requiring high costs and energy input machines. In conclusion, at the industrial scale, the recycle technology to process PPW could reach a large potential market, resulting in being more attractive for consumers. However, the challenging task to be addressed is to overcome specific technological limits in order to maximize the yield reducing costs, energy, and solvent consumption.

### 2.3. Molecular Approaches to Modulate Eco-Friendly Bioprotector Production in Potato Peel

**Figure 2:**
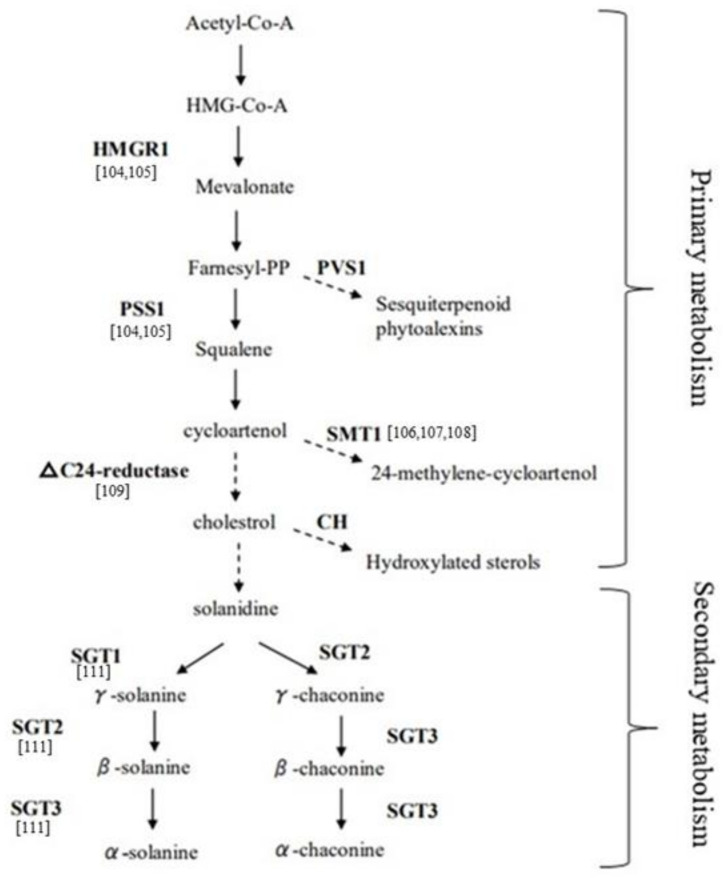
Glycoalkaloid biosynthetic pathway in potato (modified from Khan et al.) [102].

Addressing the genetic basis that influences SGAs content and composition is a key challenge to exploit PPW as bioprotector. Genome-wide association mapping studies and marker-assisted selection to find QTLs (quantitative trait locus) and markers involved in the trait have been reported [100], even if the expression pattern of SGA biosynthesis genes to be employed in breeding programs should be better understood. The biosynthesis of SGAs has been highlighted to be via the mevalonate/isoprenoid pathway (Figure 2) [101,102]. The key enzymes involved in the mevalonate biosynthetic pathway, from the top to the bottom, are, respectively, HMRG1 (3-hydroxy-3-methylglutaryl coenzyme A reductase 1) and PSS1 or SQS (Squalene synthase), that act at the pre-cycloartenol part and for which a coordinated regulation is known [103]. The *PVS1* (*Vetispiradiene sequiterpene*) gene facilitates the branching pathway that converts the farnesyl-PP into sequiterpenoid. The genes leading to solanidine from sterol precursors, including cholesterol, are *∆^24^-reductase*, *STM1* also named *SMT1* (*Sterol C24-methyltransferase* type 1), and *CH* (*Cholestrole hydroxylase*). Finally, SGT1 (*Solanidine galactosyltransferase*), SGT2 (*Solanidine glucosyltransferase*), and SGT3 (*Glycosterol rhamnosyltransferase*) represent key enzymes in the biosynthesis of solanine and chaconine. The role of most of these enzyme encoding genes has been studied in transgenic potato lines. Ginzberg proposed a feedback regulation cycle in transgenic plants where over-expression of *HMG1* resulted in increased transcript levels of endogenous *PSS1*, and, consequently, the over-expression of *PSS1* caused a reduction of *HMG1* transcript level [104]. Several studies on the characterization of the *STM1* gene have been reported. Over-expression of *STM1* led to an increase of sterol level, due to an increased flow of sterol precursors into the 24-alkylated pathway, and to a reduced cholesterol and glycoalkaloid content in leaves and tubers, suggesting that cholesterol is a precursor for the glycoalkaloid biosynthesis [105]. Factors such as wounding, light, and microbial pathogens exposure have been demonstrated to be regulators of SGA synthesis, via modification of STM1 activity. In particular, down-regulation of *STM1* acts as a fungal elicitor, affecting sterol synthesis by down-regulation of the *PSS1* gene [106,107]. A study reporting the down-regulation of the *∆^24^-reductase* in transgenic lines resulted in low levels of cholesterol and glycoalkaloids, demonstrating the involvement of this gene in the SGA pathway [108]. Moreover, another key enzyme involved in the biosynthesis of cholesterol and related SGAs, the *Sterol Side Chain Reductase 2* (SSR2) that acts between C-24 alkylsterols and cholesterol steps, has been identified and characterized in potato by genome editing [109]; in fact, loss of function of this gene showed highly reduced levels of cholesterol and SGAs in plants. Tubers genetically modified to reduce the glycoalkaloid content, via down-regulation of the three genes *SGT1*, *SGT2,* and *SGT3* acting at the end of the SGA pathway, resulted in significant changes in specific glycolalkaloids compared to wild type [110]. Moreover, a compensatory effect has been described, when the *SGT1* gene was down-regulated, α-solanine accumulation was inhibited whereas α-chaconine content increased; conversely, when SGT2 was down-regulated, α-chaconine was lowered compared to α-solanine, which increased. On the other hand, down-regulation of *SGT3* lowered both above-mentioned glycoalkaloids to varying degrees.

Natural variation in candidate SGA-related biosynthetic genes and whole-genome genotyping represent a good strategy to determine the relationship between genes and SGA accumulation. A research focusing on five candidate genes related to the primary (*3-hydroxy-3-methylglutaryl coenzyme A reductase* 1 and 2, *HMG1* and *HMG2*; *2.3-squalene epoxidase*, *SQE)* and the secondary metabolism (*SGT1* and *SGT2*) has highlighted a higher level of polymorphism in introns than exons and in genes of the secondary metabolism compared to the primary [111]. In addition, haplotypes of informative single -nucleotide polymorphisms (SNPs) in these candidate genes able to discriminate among high, intermediate, and low levels of SGAs in wild potatoes have been identified, therefore useful as functional markers to test segregating populations or association mapping panel. Similarly, Hardigan used a panel of 67 genotypes to capture the genome variation in cultivated and wild potatoes, by analyzing the sequence and the structural variants in the form of SNPs and copy number variation (CNV), against the reference genome of *S. tuberosum* Group Phureja [112]. Signatures of selection have been observed in *squalene synthase (SQS)* and in *glycoalkaloid metabolism 9 (GAME9),* an *APETALA2/Ethylene* response factor, being candidates for landraces and cultivars. Moreover, *GAME9* was located on chromosome 1, where a highly significant QTL was reported, explaining a major proportion of the SGA content in potato tubers [113]. The region spanning 230 kilobases pair included many transcription factors together with *GAME9*. Transcriptomic and proteomic analyses are important tools to provide insight into the regulation of SGA biosynthesis. Understanding regulatory players of response to environmental stress conditions can be useful in breeding and quality assessment in order to predict the levels of SGAs expression. Tubers of two potato cultivars, characterized by low (cv. Atlantic) and high SGAs content (cv. Haryoung), showed a very different transcript accumulation of *HMG1* and *PSS1* genes, under drought stress conditions, being doubled in the cultivar Haryoung [114]. The mRNA level of these two genes could be used as selection markers for breeding potatoes with low SGAs level. In addition, the abundance of transcripts of *SGT* genes was also detected, indicating their direct involvement in glycoalkaloid accumulation. A transcript profiling on two different cultivars during glycoalkaloid-inducing treatments (wounding and light exposure) has also been carried out and only a small number of differentially expressed genes, covering important steps of the entire SGAs biosynthetic pathway, was found to be associated with increased SGAs levels [115]. In particular, four genes (*MVD, Mevalonate diphosphate decarboxylase*; *FPS2, Farnesyl diphosphate synthase 2*; *SMO1-like, Sterol C4-methyl oxidase 1-like*; *DWF1-like, Sterol ∆^24^-reductase-like)* were found to be strongly induced upon wounding, whereas *MVD* and *FPS2* resulted in being not up-regulated under light exposure. The differences in the expression of these genes underlies the existence of cultivar variations in basal SGAs levels. A relation between SGAs content and the expression of *GAME*, *SGT1,* and *SGT3* genes was also reported in potato tubers by Mariot et al. [116]. A detailed analysis of *GAME*, *SGT1,* and *SGT3* promoter regions highlighted *cis*-elements related to the response of potato plants to biotic and abiotic stresses, confirming that unpredictable variations in SGA levels could be related to these stressors. Indeed, diverse studies also reported that higher levels of SGAs can impart strong resistance against pests [36,117]. In particular, Zhang et al. investigated the gene expression profiles of SGAs, induced by light exposure, in potato tubers under biotic stress [118]. A strong correlation between the stress response and SGA accumulation has been found, with both disease resistance and SGA biosynthesis genes resulting in being up-regulated. A study aimed to elucidate the defense response activated by BABA (β-aminobutyric acid), known to induce resistance in a wide range of plants against several types of pathogens, highlighted the down-regulation of genes involved in sterol biosynthesis and up-regulation of sesquiterpene phytoalexin biosynthesis enzymes, whereas a high level of pathogenesis proteins was accumulated [119,120]. Another example of correlation between SGA biosynthesis and plant defense against biotic stresses has been reported in two lines of *Solanum tuberosum* in which the *Glycoalkaloid metabolism 4 (GAME4)* enzyme encoding gene involved in the conversion of cholesterol to SGA aglycones was silenced by RNA interference [121]. When exposed to insect pest CPB (Colorado Potato Beetle), a gregarious defoliator for the *Solanaceae* plant family, inoculated with *Verticillium dahliae* pathogen, *GAME4* RNAi lines showed changes in metabolite profile, including increased levels of phytoecdysteroids, thus affecting the growth of the insect pest and, in one of the two lines, also affecting the colonization by the pathogen. Taken as a whole, these results demonstrate that in potato, targeted modifications of secondary metabolic SGA pathways can affect plant disease resistance.

In conclusion, in order to increase extraction yield from PPW and contribute to its full potential of economic value both as a by-product and as. a crop bioprotector from biotic stresses, the development of genotypes with a high level of SGAs in peel, via breeding or New Breeding Technologies (NBTs), should be a future perspective.

## 3. Biocompounds in *Brassicaceae*

Plants of the *Brassicaceae* family are characterized by a very effective and specific chemical defense system based on the production of a group of secondary anionic metabolites called glucosinolates (GSLs) [26]. GSLs consist of a S-β-d-glucopyrano unit anomerically connected to an O-sulfated (Z)- thiohydroximate function, which represents the invariant backbone of the molecule, which is linked to a variable side chain (or R-Group). More than 130 GSL structures have been discovered and validated in the whole Brassicales order of plants to date [121]. They are most abundant in species belonging to the *Brassicaceae* family such as mustards, broccoli, cauliflower, cabbage, and the model plant *Arabidopsis thaliana* [122,123].

### 3.1. Glucosinolates, Myrosinases, and Hydrolysis Products

**Figure 3:**
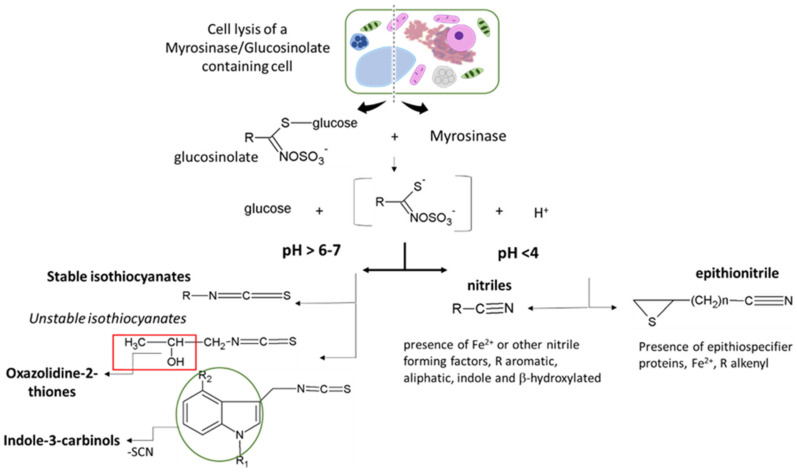
Glucosinolate hydrolysis products reactivity according to the structure of the glucosinolate side chain (R), the presence of supplementary specifier proteins, and/or the physiochemical reaction condition.

GSLs are not toxic per se but become biologically active upon hydrolysis by myrosinases and associated proteins to form GSL hydrolysis products (GHPs). In plants, GSLs and myrosinases are compartmentalized in different tissues or different parts of the same cell and get together only after tissue disruption leading to the formation of several hydrolysis products characterized by different physicochemical properties and biological activities [6]. The myrosinase-catalyzed reaction starts with the cleavage of the thioglucosidic linkage, resulting in the release of a D-glucose and an unstable thiohydroximate-O-sulfate, known as aglucone, that undergoes “Lossen-like” rearrangements and degradation to afford a wide range of hydrolysis products [121]. The outcomes of the unstable aglucone have been shown to depend on (i) the structure of the GSL side chain, (ii) the presence of supplementary proteins known as specifier proteins, and/or (iii) the physiochemical reaction condition (Figure 3). Several myrosinases have been characterized from more than 20 species of Brassicales, insects, and many bacteria residing in the human intestine. Plant myrosinases are reported to be generally activated by ascorbic acid, while in insects and bacteria, myrosinases seem not to be influenced or inhibited from it. This highly regulated defense system has been continuously updated during the plant and herbivore evolution paths. Plants evolve to defend themselves against insects by producing many different chemicals that are toxic to herbivores and other pests, and the latter ones evolve to defend themselves through detoxification pathways [124,125]. As a consequence, some plants have gained the ability to produce more than one type of chemical defense. In the *Brassicaceae* family, wallflowers of genus *Erysimum*, for example, produce two types of toxic chemicals: Hydrolysis products of GSLs, and cardenolides, which are otherwise found only in distantly related plants such as foxglove and milkweed. The combination of these two chemical defense compounds within the same plant may explain the evolutionary success of this genus within the last 2 million years [125].

### 3.2. Brassicaceae Eco-Friendly Plant Bioprotector Activity against Biotic Stresses

A role of GLs in constitutive or inducible defenses against microbial pathogens and insect herbivores has been reported [126], as they act as signaling molecules, and may initiate pathways such as stomatal closure, apoptosis, and callose accumulation [127,128].

**Figure 4:**
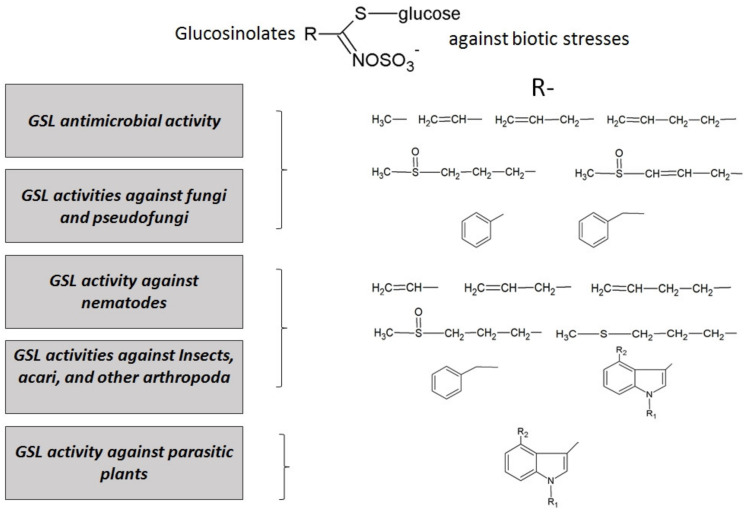
Main activities of glucosinolates and their hydrolysis products against biotic stresses in plant protection. The side chains (R) of glucosinolates, studied to date for each effect, have also been reported.

The action of these compounds against biotic stresses suggests that external application of GSLs could promote plant resistance as an alternative to chemical pesticides. The most effective GSLs have been searched by testing them in vitro against different pathogens, in order to proceed to develop new formulations for field treatments. Strong effects of GSLs have been found against many biotic agents, from bacteria to herbivores, and they are summarized in Figure 4.

#### 3.2.1. GSL Antimicrobial Activity

A clear effect on certain bacteria has been shown for different specific GSL compounds. Indeed, when the antibacterial activities of 4 isothiocyanates (ITCs) (3-butenyl, 4-phentenyl, 2-phenylethyl, and benzyl isothiocyanate) were investigated in vitro against four Gram-positive bacteria (*Bacillus cereus*, *Bacillus subtilis*, *Listeria monocytogenes*, and *Staphylococcus aureus*) and seven Gram-negative bacteria (*Aeromonas hydrophila*, *Pseudomonas aeruginosa*, *Salmonella choleaesuis*, *Salmonella enterica*, *Serratia marcescens*, *Shigella sonnei*, and *Vibrio parahaemolyticus*), benzyl and 2-phenylethyl isothiocyanate (2-PEITC) showed higher activity against most of the pathogenic bacteria than 3-butenyl and 4-pentenyl isothiocyanate, and were more effective against Gram-positive bacteria than against Gram-negative ones [129]. For these properties, GSL-myrosinase system has also been employed to prevent bacterial and fungal (see below) spoilage and to improve shelf life of food product in advanced packaging systems [130].

#### 3.2.2. GSL Antifungal Activity

Other molecules have been shown to be characterized by a wide spectrum of activities, as in the case of some *B. carinata* GSL-derived ITCs, which were significantly effective in vitro control of four assayed pathogens seedborne fungi tested by Pane et al. [131] (*Alternaria dauci, Alternaria radicina, Colletotrichum lindemuthianum,* and *Ascochyta rabiei*), confirming their high antimicrobial activity [132]. Recent experimental evidence showed that ITCs were also promising for treatments against black spot, one of the most important diseases of pear fruit during storage caused by *Alternaria alternata*. The development of black spot rot on the pear fruit inoculated with *A. alternata* was significantly decreased by 2-PEITC fumigation [133]. The authors proposed that the antifungal effect of 2-PEITC against *A. alternata* might be mediated by a reduction in toxin content and breakdown of cell membrane integrity. A similar effect on fungal toxins has been observed for prop-2-enyl or allyl isothiocyanate (AITC)s in the treatment of post-harvest products such as in maize. Nazareth et al. stated that AITC caused a transcriptional alteration of genes involved in aflatoxin B1 and other processes key for normal fungal growth and development [134,135]. The authors reported prevention of the growth and development of *A. niger, A. parasiticus,* and *Fusarium verticillioides*, as well as the reduction of mycotoxins (aflatoxins and fumonisins) by GHPs application. Additional studies showed that gaseous AITC, benzyl, and phenyl isothiocyanate inhibited the growth of different fungal species and the production of mycotoxins in in vitro studies and food products [136,137,138]. Similar effects, starting from flours of oriental and yellow mustard, were obtained for wheat tortillas as preservatives against aflatoxins B1, B2, G1, and G2 [139]. Numerous data reported in the scientific literature encourage the use of GSLs to develop new formulations for the sustainable protection of crops from abiotic stresses, and a similar strategy can be imagined also for other cereals crops.

#### 3.2.3. GSL Activity Against Nematodes and Insects

Eleven GSLs and their degradation products were evaluated for the biocidal activity of on second-stage juveniles of the root-knot nematode *M. incognita* in vitro [140]. None of the intact GSLs showed any biological effect. Following myrosinase addition, GHPs (essentially ITCs) resulted in highly different biocidal activities. Similarly, Bhushan et al. showed the insecticidal potential of 3-isothiocyanato-1-propene or AITC on the growth and development of a polyphagous pest, *Spodoptera litura* (Fab). AITC resulted in 100% larval mortality when applied at the highest concentration (3125 ppm) [141]. Larval period, pupal period, total development period, and pupal weight were also influenced by AITC. Wireworms are widely distributed throughout the world and are important pests of a wide range of crops including small and large grain cereals. *Brassica carinata* defatted seed meals (DSMs) used on maize plant at early development stage in pots and in open field experiments showed levels of wireworm control and prevention of plant damage comparable to the conventional insecticide (Regent^®^, BASF Italia [142]. In these trials, the efficacy of AITC, the main GHPs in *B. carinata* was tested; in literature several applications especially for maize, are reported. Indeed, AITC was recently proposed as a potential tool to be employed in control strategies against maize weevils, to overcome resistance to phosphine and other conventional insecticides [143,144]. Other Brassicales, such as *Eruca sativa* and *Brassica rapa* seed powders, showed allelopathic activities due to the presence of different GSLs, as selective bioherbicides for controlling *Cyperus rotundus,* improving both growth parameters and carbohydrate contents in maize cultivation [145].

As for the interaction with the herbivores, a high level of indole GSLs had a positive action against pea aphids (*Acyrthosiphon pisum*) but not against peach aphids (*Myzus persicae*) [146]; the last mentioned study showed that GSLs may have both direct and indirect effects on dodder-feeding herbivores.

#### 3.2.4. GSL Activity Against Parasitic Plants

The effect of GSLs is not limited to microorganisms and animals. Recently, an interesting study by Smith et al. investigated the effect of GSLs on a parasitic plant and on the herbivores that grow on it [146]. Transgenic *Arabidopsis* lines with elevated indole GSLs or without them, as control, were developed and allowed to interact with the parasitic dodder vines (*Convolvulaceae*; *Cuscuta gronovii*). Parasitic plants acquire diverse secondary metabolites from their hosts, including defense compounds that target insect herbivores; in this case, concentrations of aliphatic and indole GSLs were higher in parasite tissues than those observed in corresponding host tissues. Dodder growth was enhanced on plants without indole GSLs and inhibited on plants with elevated indole GSLs compared to wild-type hosts. Therefore, an additional defensive role of indole GSLs against parasitic plants can be argued.

#### 3.2.5. GSLs Role in Biofumigation Crop Protection Management

Biofumigation is a sustainable agronomic practice for pest management based on the release of volatile hydrolysis products of GSLs. It involves the sustainable and circular use of renewable and biodegradable plant materials, determines a significant reduction of CO_2_ emissions, returns organic matter to the soil, and is less toxic to the general soil environment than synthetic pesticides. It can be achieved through several methods. One of the most beneficial practices for soil health is the green manure technique, often coupled with appropriated crop rotations, which consists in growing selected *Brassicaceae* as a cover crop and tilling them into the soil where they break releasing by-products of GSLs. Biofumigation tissues or bio-based products may also be applied as industrially formulated DSM or concentrated plant essential oils or extracts [147,148,149,150,151].

The agronomic technique of biofumigation is based on the idea that *Brassicaceae* tissues may sustain the molecular defense system of a profitable crop providing it with peculiar hydrolysis products of GSLs. In fact, at least since 2005, when the process of phasing out from agriculture of methyl bromide occurred in all the major countries [152], several non-synthetic chemical alternatives have been explored in order to substitute this widespread fumigant. Among these low-impact alternatives, the biofumigation techniques have established and widespread in the last 30 years. The first applications were plants, selected for rusticity, biomass yield, and concentration of specific GSLs in epigeal and/or hypogean tissues, used as intercrops or biofumigant green manures [153,154]. Afterwards, beside plants, seed- and plant-derived materials were optimized to release allyl-isothiocyanate. In fact, *Brassica* plants translocate GSLs in the seeds at a high concentration, becoming a starting material for biofumigant bio-products. After seed defatting, the residual meal contains a high level of GSLs. The obtained meal, or pellet, can be formulated by a patented procedure able to modulate ITCs release [155,156]. *Brassica* crops or seed meal amendments incorporated into soil may have the potential to control soil-borne plant pathogens, by changing soil pH, microbial populations, and enhancing enzymatic activities concurrently with the release of ITCs [157]. These processes may trigger a new microbial balance manifested as soil suppressiveness, that is “soil’s ability to delay pathogen infection and disease progress in a susceptible host, even in the presence of virulent pathogens”. In fact, even when less effective, different *Brassica* treatments—both green manure and seed meal—either improve or maintain soil microbial activity and fertility compared to the chemical treatments [158]. Generally, meals and pellets are distributed on dry soil, incorporated at 20–30 cm depth at a rate of 250/300 g m^−2^ and activated by light irradiation. In this way, the meal releases ITCs directly into the soil limiting significantly active compound losses in the air by volatilization. Afterward, liquid formulations based on a *Brassica* oil in water emulsion with the addition of a reduced amount of biofumigant meals were conceived. In this case, GSL hydrolysis is activated by the water in the emulsion, and, according to its hydrophobicity, the released ITCs are solubilized in the oil fraction of the emulsion. Liquid emulsion, once distributed, forms an oil microfilm on the plant organs, determining a potential repellent effect and a physical suffocating action on some pests and pathogens, meanwhile improving the biofumigant effect of ITCs [159].

In Table 2, the experimental evidence mainly reported in the last 10 years of literature about the molecular system of *Brassicaceae* involving GHPs released by the GSL/myrosinase system, including a clearly or tentatively defined GSL concentration in experimental trials and the plant pathogens involved in the studies, is summarized. Table 2 [160,161,162,163,164,165,166,167,168,169,170,171,172,173,174,175,176,177] shows the growing and keen interest for biofumigation applications, particularly as regards plant protection against nematodes, fungi, pseudofungi, and some arthropoda. Nevertheless, soil organic matter addition through plant-based products should be carefully evaluated. As an example, even if in vitro assays, containment of *Fusarium* spp. through AITC was established [124], and the control effect on *F. graminearum*, a severe wheat pathogen, was demonstrated in the field condition [178]. Several doubts are still raised on *Brassicas* applied as green manure, given their host status for some *Fusarium* species, for example some *formae speciales* of *Fusarium oxysporum* known for being pathogenic on *Brassica* crops that become a vehicle for increasing infection and some non-reproducible results [179]. Despite their effectiveness, Brassicales-based products’ application in commodity crops is still underestimated. This is principally due to the economic costs, which are rather high to date. On the other hand, as already mentioned, future EU strategy will lead to a progressive reduction in chemicals applied in agriculture. This process already begun years ago, since many of the high-impact chemical products used in European agriculture are being phased out (Directive 2009/128/EC) [5], and non-chemical alternatives are strongly needed as suggested by Regulation (CE) no.1907/2006 (REACH) [180]. Furthermore, the secondary effects of such products have been underestimated. In fact, besides their biological effects, most *Brassica* DSMs are characterized by a valuable level of Nitrogen [181] with a good C/N ratio, making them suitable soil amendments. Again, in the near future, bio-product environmental impacts in terms of CO_2_ sequestration or, more in general, their LCA (life-cycle assessment) should be considered. Besides the suitable C/N ratio, a *Brassica*-based formulated product could consistently reduce CO_2_ emissions [25], while still being effective and less harmful to the soil food web functioning [120,162] and to the beneficial soil invertebrate [177], thus reducing agricultural environmental impact [156].

### 3.3. Molecular Approaches to Enhance GSL Content in Brassicales

GHPs from aliphatic, indolic, or aromatic are associated with diverse phytochemical activities spanning from beneficial effects for agriculture (pest management and biofumigation) to nutraceutical properties when brassicaceous vegetables are part of human diet. Understanding GSL properties has led to the identification of molecular target for both classical breeding and biotechnological approaches to specifically enhance the content of GSLs and thus address a specific trait of interest. GSL profiles vary significantly among *Brassicaceae,* and GSL synthesis is a complex quantitative trait; the molecular basis of these variations is largely unknown. The history of GSL research can be seen as a sort of “opposite sides of the same coin” since the identification of targets for agricultural improvement moved in parallel with findings on GSL phytochemical properties. Indeed, in the first instance, the main target of GSL research was to reduce their content in a specific tissue or in the whole plant, thus reducing the goitrogenic effect and use Brassicales-derived seed cake as animal feed [182]. To achieve this goal, researchers exploited germplasm biodiversity, first finding the *B. napus* L. variant with low erucic acid (the 0-variant) and later on the Bronowski variety (the 00-variant) with both low erucic acid and GSLs. The latter variety is nowadays used as genetic background for the *B. napus* cultivars employed in agriculture [183]. Conversely, once the GSL beneficial effects for both human and plants were discovered, the research goal shifted from lowering the content of GSLs to increasing it. In particular, certain GHPs like sulforaphane or 4-(Methylsulfinyl)butyl ITC, which is the isothiocyanate derivative of 4-(methylsulfinyl)butyl GSL found in broccoli, have been identified as potent cancer-preventing agents and for this reason, increasing their content has been the focus of many studies in the past decade.

Wild relatives are valuable sources of desirable quality traits that can be subjected to introgression via breeding into elite cultivars, developing new and improved crop varieties. The *Brassicaceae* family encompasses many important crops [184] and includes the model species *Arabidopsis thaliana*. Wild relatives of the *Brassica oleracea* species have been shown to be characterized by high levels of aliphatic GSLs whose analysis revealed candidate taxa for broccoli breeding programs aiming at specifically increasing the concentration of 4-(methylsulfinyl)butyl GSL. An example is represented by members of *Brassica villosa-rupestris* with a non-functional *GSL-ALK* allele and thus can be considered as the progenitors of cultivated broccoli [185]. The beneficial effect of cruciferous-derived food products was the main target of classical breeding programs aiming at increasing the concentration of glucoraphanin, an aliphatic thiofunctionalized GSL. Indeed, an ITC-enriched broccoli variety, called Beneforté and nowadays commercialized in the UK, was obtained through introgression of three QTLs from the wild relative *B. villosa* to the commercial variety *Brassica oleracea* var. *Italica* via marker-assisted selection (MAS) [186]. Interestingly, this variety is commercialized with a health claim addressing the beneficial effects of cruciferous-derived food products. An additional example of a successful breeding program is provided by Li and co-workers, who obtained segregating populations of *Brassica oleracea* L. by three crosses: Broccoli × cauliflower, collard × broccoli, and collard × cauliflower [187]. These segregating populations showed a high content of 4-(methylsulfinyl)butyl GSLglucoraphanin and a low amount of anti-nutritional GSLs, such as (R)-2-hydroxybut-3-enyl GSL progoitrin, thus selectively modifying the aliphatic GSL composition. Furthermore, these recombinant inbred lines allowed to better elucidate the functional role of *A. thaliana* ortholog genes, *GSL-ELONG, GSL-PRO, GSL-OH,* and *GSL-ALK,* which are involved, respectively, inside chain elongation, hydroxylation, and secondary modification. Indeed, non-functional *GSL-OK* or *GSL-ALK* alleles were responsible for the observed biochemical GSL profile. Based on this study, Liu et al. [188] used an RNAi strategy to knockdown the expression of *GSL-ALK* gene thus obtaining *B. napus* lines with a strong reduction of (R)-2-hydroxybut-3-enyl GSL progoitrin and an increase of 4-(methylsulfinyl)butyl GSL glucoraphanin. Based on the assumption that amino acids like methionine and phenylalanine boost the synthesis of aliphatic and aromatic GSLs, respectively, strategies aiming at increasing their availability have been successfully addressed [189].

#### 3.3.1. Biotechnology Approaches to Enhance GSL Content

Genetic engineering technologies have been applied to modulate GSL profiles via heterologous expression of CYP79 enzymes. Indeed, Brader and co-workers expressed the *CYP79* transgenes into *A. thaliana* plants [190]. Transgenic plants expressing the CYP79D2 showed high amounts of isopropyl and methylpropyl GSLs and resistance to *Erwinia carotovora*, a bacterial pathogen. *A. thaliana* plants over expressing CYP79A1/A2 were less susceptible to *P. syringe* but more susceptible to *A. brassicicola* suggesting that different GSLs act differently as pathogens’ defense, and this may be due to their specific modes of action. Wentzell et al. demonstrated that the *AOP2* enzyme encoding gene is the major regulator of aliphatic GSL biosynthesis and accumulation; furthermore, its overexpression resulted in upregulation of the entire biosynthetic pathway [191,192]. The recent release of *Eruca sativa* genome [193] will represent a valuable resource to identify the gene target for genetic engineering strategies in this species. Elicitation, through diverse approaches both physical and chemical, is an experimental procedure well established to modulate the content of secondary metabolites in plant tissues/organs and examples about GSLs were also recorded. Due to the fact that methionine-derived GSLs are sulfur-rich metabolites, sulfur fertilization affects their concentration leading to an increase in the range of 25–50% depending on (*i*) the plant species, (*ii*) amount of sulfur applied, and (*iii*) type of treatment. The higher concentration of GSLs is related to upregulation of the entire biosynthetic pathway; conversely, sulfur-deficient plants showed a down regulation of GSL biosynthetic genes [194].

Taking advantage of knowledge of the biosynthetic pathway leading to these secondary metabolites, mainly derived from research on *A. thaliana*, several strategies have been applied to engineer enzymes encoding genes in the first instance, and only upon the discovery as master regulator was the *MYB28* transcription factor was addressed too [195,196]. Traka and co-workers [196] found that the high-glucoraphanin4-(methylsulfinyl)butyl GSL F1 broccoli hybrids developed via an introgression breeding program with *B. villosa* showed a high induction of *MYB28* allele from the wild *B. villosa* suggesting its role in regulating the expression of glucoraphanin4-(methylsulfinyl)butyl GSL biosynthetic pathway components.

The use of plant tissues, hairy roots cultivation, and cell cultures, even in the presence of elicitors, may represent an efficient plant platform for the production of active metabolites [197] in a plant molecular farming scenario. Despite several attempts, the use of cell suspension culture for GSL production did not lead to successful yields compared to *in planta* content highlighting that it is not a suitable strategy for large scale production [189]. Tissues/organ and liquid cells culture mainly from *A. thaliana* var. Col0 were exploited by using both metabolic engineering and elicitation (e.g., hormones supply) approaches. The hairy root system has been widely used in aromatic plant species to specifically target and scale-up the production of phytochemically relevant metabolites. Kastell et al. [198] used this system to express the transgenes encoding for entry point enzymes of the. aliphatic pathway, *CYP79F1* and *CYP79F2* genes; however, the overall GSL rate was very low compared to leaf tissue. Studies combining elicitor treatments have also been conducted; acetylsalicylic acid in combination with phenylalanine and cysteine amino acids resulted in being the best elicitors among tested hormones, inducing an increase of benzyl GSLs [199]. Table 3 shows the biotechnology approaches to enhance GSL content mentioned herein.

#### 3.3.2. GSL Molecular Markers and Gene Mapping

Based on *A. thaliana* GSL-related gene sequence information, a positional cloning strategy was applied to identify orthologs in *B. oleracea* genome. The *B. oleracea* genes *BoGSL-ELONG* and *BoGSL-PRO* together with *BoGSL-ALK* and *BoGSL-OH* were mapped on a high-density *B. oleracea* linkage map [202], which may be part of a QTL region for GSLs [203]. QTLs were identified in *B. rapa* too by means of comparative genomics and synteny with *A. thaliana* [204,205]. Biosynthetic genes identified in *B. rapa* and QTLs were reported [206]. A recombinant inbred line (RIL) population was developed from crossing Chinese Cabbage *(Brassica rapa* L. *chinensis*) and yellow sarson (*Brassica rapa* L. subsp. *trilocularis*) and used to fine map seed GSL trait in *Brassica rapa* L. [207]. Taking advantage of a previously reported ultra-dense genetic map of *B. rapa*, molecular markers associated with GSLs gene and SSR were found, and a QTL analysis was performed. The authors reported the identification of a major QTL for 4C and 5C GSL, which colocalized with *GSL-ELONG* locus *SCAR* marker *BrMAM1-1* in *B. rapa* seeds, thus identifying a candidate gene, *Br- GSL-ELONG*, for 5C side chain elongation of aliphatic GSL in this species. Recently, Zhang et al. [208] identified a major QTL controlling aliphatic GSL accumulation in *B. rapa* leaves. The QTL, which encompasses three tandem *MAM* genes and two *MYB* genes, was detected in two BC2DH populations [208]. Interestingly, the authors reported that the *BrMAM-3* gene was correlated with the accumulation of aliphatic GSLs in *B. rapa* leaves. Furthermore, a naturally occurring insertion within exon 1 of *BrMAM-3* causing a loss of function, mutation was associated with the low GSL content in *B. rapa* accessions, thereby this gene is a good candidate to manipulate aliphatic GSL in *B. rapa* via metabolic engineering and classical breeding approaches. QTLs and associated molecular markers for GSL were also discovered for amphidiploid *Brassicaceae* species like *B. napus* [209,210] and *B. juncea* [211,212]. Based on QTLs analysis, five loci on A2, A9, C2, C7, C9 *B. napus* chromosomes were associated with GSLs in seeds [212,213] while five other QTLs explain 30–45% of the total aliphatic GSL variation in *B. juncea* [214]. Zou and co-workers [215] identified five QTLs associated with GSL variance in roots of two F2 populations of *Raphanus sativus*, thus opening new breeding perspectives to enhance GSLs in a different genus. It is well established that the GSL profile is greatly variable between species, within species, and individual cultivars, thus varietal selection and development of breeding lines with uniform GSL profiles will contribute to fix plant breeding materials. Furthermore, another source for GSLs accumulation traits in breeding programs is represented by Gene bank accessions including wild genotypes, which are underutilized. Phenotyping these large germplasm collections will again contribute to identifying individual pre-breeding materials useful for GSL content improvement. Despite that GSL content and profile in *Brassicales* is influenced by several factors related to both environment and cultivation conditions, a wide genetic variation does exist.

GSL content has a quantitative genetic inheritance, regulated by complex genetic factors and affected by environment [204]. Genetic and genomic resources also have to be developed for *Brassicaceae* species not deeply studied, like rocket, radish Chinese cabbage, and *B. carinata*; there is an urgent need to identify species-specific SNPs associated with GSLs and GHPs as well as molecular markers to assist selection for highly beneficial GSLs. A successful example of MAS for GSL increase was provided by cabbage lines derived from a cross between *B. rapa* and *B. oleracea* [216]. Furthermore, a genetic linkage map and QTL analysis revealed seed quality traits like erucic acid content and GSL in *Sinapis alba* L., providing useful tools for breeding strategy mediated by molecular markers associated with GSLs components [216]. Recently, a CRISPR/Cas9-mediated editing of *MYB28* gene in *Brassica oleracea* has been achieved by Neequaye et al. [201], and although the paper has not been subjected to peer review process so far, this result shed light on the opportunity that new biotechnological approaches could be applied to modify the content of GSLs *in planta*.

## 4. Perspectives

Pollution problems in the environment and the toxic effects of synthetic chemicals on non-target organisms have prompted investigations on exploiting pesticides of plant origin. Natural plant products and their analogues resulted in being an important source of new agricultural chemicals [217,218] used in control of insect pests [219], plant diseases [9,10,220,221,222,223,224], and as a bird repellent [225,226,227]. Several studies have shown the importance of natural chemicals as a possible source of non-phytotoxic, systemic, and easily biodegradable alternative pesticides [228,229].

Plants constitutively accumulate compounds that may be involved in several different physiological roles, such as defense against pathogens and/or involved in regulatory and developmental processes [230,231,232,233]

Such antimicrobial bioactive peptides or compounds could be either toxic or inhibitory against pathogens, acting against bacteria and/or fungi and/or pests and may be expressed in the plant constitutively or induced following infection [234,235]. In their long association with pathogens, plants evolved an intricate and elaborate array of defensive tools acting in constitutive and/or inducible defense strategies, including pathogenesis-related proteins, antifungal proteins, and secondary metabolites [232,236,237]. The availability of plant-derived compounds with antifungal activities sufficient to make them feasible for agronomic use in disease control still remains inadequate to increasing environmental requirements.

In this perspective, the use of bioactive molecules derived from *Brassicaceae* defatted seed meals and from *Solanaceae* waste (peel potato recovery) for cereal crop protection could be a successful strategy with the main goal to find out new formulations of eco-friendly products with fungicidal/fungistatic/insecticidal properties. In particular, the circular utilization of Brassicaceous and Solanaceous industry waste derived by-products for disease biocontrol of (*Zea mays* L.) and wheat (*Triticum aestivum* L.) could meet the criteria of benign environmental profiles and low toxicity to humans and wildlife, with specific attention to the sustainability of cultivation and the quality of products.

In fact, mycotoxigenic fungal diseases strongly limit crop yield (up to 20% annual cereal loss) [238,239], quality, and safety, especially in maize and wheat, two of the most important sources of food all over the world with a global production of 1100 10^6^ and 129 10^6^ tons, respectively (FAOSTAT 2019). Fungi of the genus *Fusarium* and *Aspergillus* are widely distributed maize pathogens, causing diseases to occur on seedlings, roots, stalks, ears, and kernels, affecting grain quality through the production of mycotoxins, including fumonisins produced by *Fusarium verticillioides* [240] and aflatoxin B1 produced by *Aspergillus flavus* [241]. As described for maize, *Fusarium*, specifically *Fusarium graminearum*, also threatens wheat grain yield and safety due to the production of mycotoxins (deoxynivalenol-DON, zearalenone-ZEA). Mycotoxins are secondary metabolites produced by fungi, which may be toxic or have other debilitating effects on living organisms [242,243]. Bioactive molecules extracted from *Brassicaceae* defatted seed meals and from *Solanaceae* waste (peel potato recovery) should be preliminary evaluated in in vitro bio-assays for their efficacy against *A. flavus*, *F. verticillioides, F. graminearum* growth, and mycotoxin production [229,244] with the aim to highlight the most promising formulation to be used in field trials. Maize field protection could be planned by the application of bioactive extracts to maize-developing ear at the flowering stage with the aim to control the development of mycotoxigenic fungal infections and the subsequent accumulation of mycotoxins in the grain. Mycotoxin contamination in grains is a global threat to both safety of human food and animal feed, [241,245,246]. New regulations for the allowable mycotoxin limits in food and feed have been put in place in many countries. The binding European Union regulations on toxin contamination for human consumption and recommendations for animal feeding [247,248] have forced renewed efforts in finding reliable tools and solutions for control of mycotoxin contamination.

Furthermore, infection by fungal pathogens on maize can be favored by the attack of insects such as *Diabrotica virgifera virgifera* or *Ostrinia nubilalis* [248,249] that, both at larval and adult stage, can create vehicle inputs of the fungal inoculum. Interestingly, in this perspective, the utilization of bioactive extracts from *Brassicaceae* and *Solanaceae* could also be tested as possible bioprotector for maize pest treatment. An additional wheat disease to be mentioned and that could be addressed by bioactive extracts is rust infection, which may cause up to 50% yield losses, mainly due to a reduction in biomass, harvest index, and kernels per square meter [250]. The recent emergence of new widely virulent and aggressive strains of rusts (particularly stripe and stem rust) threatens wheat production worldwide, especially under the trend of higher temperature and humidity observed in the frame of the current climate changes. The bioactive molecules from *Brassicaceae* and *Solanum tuberosum* wastes could represent an innovative tool to be applied also in organic farming systems, to address the reported rust wheat disease, and for reducing phytosanitary emergencies caused by Fusarium Head Blight disease.

## 5. Conclusions

The need for effective, suitable, and alternative crop protection tools is particularly urgent considering that recent results estimate, at a global level, the losses for wheat, rice, and maize to medium values of 21.5%, 30.0%, and 22.5%, respectively, also highlighting that the highest losses are associated with food-deficit regions with fast-growing populations, and frequently with emerging or re-emerging plant pests and diseases [251]. This trend and the consequent evolution have accelerated over the past 70 years, favoring phenomena such as the rapid adaptation of populations of pests and pathogens to used synthetic pesticides and chemical protection products.

Biodegradable pesticides derived from Potato and *Brassicaceae* wastes could have a large potential market especially in a view of an integrated, environmentally friendly, and circular agro-economy. Based on the reported comprehensive results and evidence derived from scientific literature, some relevant considerations could be highlighted in the perspective of their optimal and innovative use.

In this perspective, interesting suggestions and intriguing opportunities could take advantage from the valorization of the combined effect of SGAs and phenols. The future use of potato peel could depend on the fact that major crop protection could be achieved with mixtures of compounds at levels that individually are almost or completely inactive [71]. In the frame of a green strategy for sustainable PPW exploitation, it would be necessary to investigate the most suitable genotype to obtain an innovative by-product of agro-industrial processing, which can be applied as a plant pathogen biocontrol agent in crop cultivation. Additionally, further investigations are required to optimize the recycle technology to process potato peel waste.

The great value of bioactive molecules present in Brassicaceae, a number between 88 and 137 different GSLs, with reference only to the MYR/GSL system, could be more exploited than it is currently being studied, in the perspective of increasing development of innovative plant bioprotectors; no more than 10% of available molecules have been tested to date. Nevertheless, taken together, all the *Brassicaceae* effects reported in recent years show the great potential of *Brassica* bio-based products both in field applications and in food formulation or storage.

There is an urgent need to accelerate the development and feasibility of genetic, genomic resources, and biotechnological tools (*i*) in potato for the development of genotypes with an effective compositions of biocompounds in peel and (*ii*) in Brassicaceae not deeply studied until now like *E. sativa* and *B. carinata*, with the aim to boost research to increase the content of beneficial compounds in these species, leading, meanwhile, to sustainable and circular industrial-agronomic practices.

## Figures and Tables

**Table 1:** Potato metabolites activity against biotic stresses.

Metabolite/Extract: Glycoalkaloids	Target: Molluscs	Effect	References
α-chaconine; α-solanine	*Helix aspersa* L.	Feeding deterrence	[64]
	**Target: Protoza**		
α-chaconine; α-solanine	*Trichomonas vaginalis*	Growth inhibition	[68]
Peel extracts	*Salmonella typhimurium; Eschrichia coli*	Mutagenic activity	[30]
Peel extracts	*S.aureus (Gram +); P.aeruginosa (Gram -)*	Growth inhibition	[31]
	**Target: Insects**		
α-chaconine	*Phorimaea operculella*	Strongly related with PTM larval mortality	[45]
α-chaconine; α-solanine	*Tribolium castaneum, Sitophilus oryzae* L.	Toxic against adults	[70]
α-chaconine; α-solanine	*Trogoderma granarium*	Antifeedant activity	[25]
α-solanine	*Galleria mallonella* L.	Increased mortality of larvae; pupae and adults, decreased fertility and fecundity	[27]
α-solanine	*Myzus persicae*	Antifeedant activity, decreased fecundity, increase mortality of pupae	[27]
α-chaconine; α-solanine	*Zophabas atratus*	Decrease heart activity in pupae and adults	[27]
vegetable waste extract	*Culex quinquefasciatus, Nopheles stephensi*	Larvicidal activity	[27]
leaf extract	*Zophobas atratus, Tenebrio molitor*	In vivo cardioinhibitory activity in pupae and adults, increased mortality	[27]
leaf extract	*Leptinotarsa decemlineata, Spadoptera exigua*	Increased mortality of larvae, pupae and adults, disturbance in fertility and fecondity	[27]
	**Target: Amphibia**		
α-chaconine; α-solanine	*Frog embryos*	Impact on membrane	[74]
	**Target: Fungi**		
α-chaconine; α-solanine	*Ascobus crenulatus*, *Alternaria brassicicola*, *Phoma medicaginis*, *Rhizoctonia solani*	Effect on the fungal growth	[66]
α-chaconine; α-solanine	*Ascobus crenulatus*, *Alternaria brassicicola*, *Phoma medicaginis*, *Rhizoctonia solani*	Spore germination inhibition Fungal growth inhibition	[67]
α-chaconine; α-solanine	*Alternaria alternata, Pyrenophora teres f. teres, Pyrenophora tritici-repentis*	Effect on the fungal growth	[82]
solanidine	*Phythophtora infestans*	Inhibition of mycelial growth	[77]
**Metabolites/Extract: Phenols**	**Target: Protozoa**	**Effect**	**References**
CA; CGA	*Trichomonas vaginalis*	Growth inhibition	[68]
	**Target: Insects**		
CA; CGA	*Phorimaea operculella*	Strongly related with potato PTM larval mortality	[45]

**Table 2:** Biofumigation in vitro, in pots, and in open-field trials published in the last 10 years with defined glucosinolate concentrations.

**Glucosinolate/isothiocyanate**	**Target: Nematodes**	**Effect**	**References**
Allyl GSL from leaf flour of *Brassica macrocarpa* Guss.	*Meloidogyne* spp. root-knot nematodes, target crop: tomato in greenhouse	*B. macrocarpa* leaf flour inserted in the soil at a GSL dose of 300 µmol m^−2^ showed similar effect to Vydate 5G^®^ on root disease index, root weight, and marketable yield	[160]
GSL from the DSMs of 13 *Brassicaceae* species: *Barbarea verna*, *B. carinata*, *B. nigra*, *B. rapa B. tournefortii*, *B. oleracea* var. *acephala Crambe abyssinica*, *Eruca sativa*, *Lepidium densiflorum*, *Lepidium sativum*, *Raphanus sativus*, *Rapistrum rugosum*, and *Sinapis alba*.	*Meloidogyne incognita* in pots in glasshouse-controlled conditions, with *Solanumlycopersicum L. cv. UC82* as host plant.	Among the tested DSMs, the best results for all inoculations were achieved by *Eruca sativa, Barbarea verna* (300 µmol m^−2^ GSLs) and *Brassica nigra* (370 µmol m^−2^ GSLs), whereas the other species gave either alternate results or results not different from untreated or sunflower DSM controls. All the DSMs, including sunflower, determined a clear positive effect on tomato vigour.	[149]
Allyl GSL (98% of total GSLs) from *B. juncea*; 4-(Methylsulfinyl)butyl GSL (72 % of total GSLs) from *R. sativus*; and 4-pentenyl GSL (38% of total GSLs) from *E. sativa*	cyst nematode *Globodera pallida,* target crop: potato	10 kg ha^−1^ *E. sativa*, 8 kg ha^−1^ *B. juncea*, and 20 kg ha^−1^ *R. sativus* were sown, cultivated, and incorporated in soil, in open field trials, both in summer and winter. The incorporation of green materials was done at complete flowering for summer trials or one week prior the potato planting for winter trials. A positive linear regression of GSL concentration (mol m^−2^) in incorporated biomasses and *G. pallida* mortality was determined for all brassicaceous species cultivated during summer; only *R. sativus* and *E. sativa* demonstrated a significant relationship between GSL concentration and *G. pallida* mortality.	[161]
GSL from *Brassica carinata* DSM	*Meloidogyne incognita* root-knot nematodes, target crop: tomato in greenhouse.	A pot trial was conducted on tomato plants grown in a soil naturally infested with *M. incognita,* amended with *B. carinata* DSM (3 t ha^−1^ or 40 mmol m^−2^ GSL), and finally compared to a soil fumigated with Vapam (Sodium methyldithiocarbamate) and to an untreated control. Both *B. carinata* DSM and Vapam treatments were effective in protecting tomato plants against *M. incognita* but they exhibited different effects on soil biota. In general, nematode populations strongly responded to *B. carinata* DSM amendments both in terms of abundance and structure. Although the free-living nematode structure was negatively influenced by the two treatments, *B. carinata* DMS proved to be the best compromise between efficiency to control *M. incognita* and environmental impact.	[162]
GSL from four cultivars: three mustards (*Brassica juncea* ‘Caliente 61′, ‘Caliente 199′, and ‘Pacific Gold’) and one broccoli (*Brassica oleracea* var. *botrytis* ‘Arcadia’)	2-year open field study of biofumigant of the four Brassicales in a chile pepper *Capsicum annuum* ‘AZ-20′, rotation system in southern New Mexico.	Broccoli produced lower biomass and lower GSL concentrations than the mustard treatments but may be a valuable crop for growers with nematode issues because *Meloidogyne incognita* populations decreased in its presence. Based on high biomass production and high GSL concentration, *B. juncea* ‘Caliente 199′ showed the most potential as a biofumigant crop for southern New Mexico	[163]
Leaf flour of dry plants of *B. juncea*, *E. sativa*, *R. sativus* and *B. macrocarpa*, characterized for sinigrin content.	*Meloidogyne* spp. root-knot nematodes, target crop: tomato in greenhouse	Leaf flours were distributed before planting (60 and 90 g m^−2^), with the mean dose corresponding to allyl GSL content in the commercial formulate (Nemathorin) applied at 3 g m^−2^. Disease index detected on the tomato roots at the end of the growing cycle resulted in all thesis lower than the control and Nemathorin, whereas it was lower with 60 g m^−2^ *E. sativa* and 90 g m^−2^ *R. sativus*, in comparison to 90 g m^−2^ *B. juncea*.	[164]
**Glucosinolate/isothiocyanate**	**Target: Fungi**	**Effect**	**References**
Pure AITC, and macerated plant tissues from 18 different cultivars amongst *Raphanus sativus*, *Sinapis alba*, *Brassica carinata*, *Brassica juncea*	*Fusarium gramineaum* and *Fusarium poae* in vitro and pot experiment	*B. carinata* and *B. junceae* (AITC containing tissues) performed a better mycelial growth reduction in Petri dish. *Fusarium poae* resulted more tolerant to AITC than *F. graminearum*. In general, all pots added with *Brassicaceae* plant material presented a reduced fungal infection, but only *B. juncea* plant material alleviated *F. graminearum* negative effect on maize growth.	[165]
Pure Allyl isothiocanate	maize grains contaminated with *Aspergillus flavus* in glass jars	maize grains contaminated with A. flavus in glass jars of 1 L and treated with 0.125, 0.25, 0.5, 1 and 5 μL of AITC. After 7 days of storage, the mycelial growth was significantly reduced in doses higher than 0.125 μL/L of AITC. All doses of AITC significantly reduced the fungal growth and Aflatoxin B1 production in maize after 30 d, regardless of moisture content.	[135]
GSL derived from bio-based experimental formulations con-taining either *B. carinata* oil 1.5% and *B. carinata* DSM 3 g L^−1^ (270 µmol GSL L^−1^), or 2% *B. carinata* oil and *B. carinata* DSM 4.5 g L^−1^ (405 µmol GSL L^−1^).	*Podosphorea xanthii* control on melon in open field	The field trials carried out over two years demonstrated the efficacy of the two bio-based experimental formulations based on *B. carinata* biomasses. In particular, the formulation with the highest concentration of oil and DSM gave results statistically not different from those of penconazole (Topas).	[166]
AITC from six *B. juncea* (L.) Czern cultivars, two *B. rapa* cultivars and one *B. oleracea*, as macerated of frozen plant tissues	*Rhizoctonia solani* AG1-1A, in vitro assays. Target plant: *Oryza sativa*	3 g of macerated frozen tissues, amended or not with 3 different soils, were confined to the lid of an upside-down Petri dish, containing potato dextran agar (PDA) medium with a disc of agar inoculated with *R. solani* AG1-1A. The dishes were incubated for 72 h at 25 °C. All six *B. juncea* cultivars consistently inhibited mycelium growth (90% inhibition) in all soils tested; *E. sativa* which was not considered as a brassicaceous, achieved 60% *R. solani* AG1-1A inhibition if amended into soils with the lowest levels of pH, organic matter, proteins, calcium and magnesium.	[167]
GSLs from above-ground parts of *Brassica juncea* (L.) Czern. & Coss, ‘Negro Caballo’, *Eruca sativa* Miller, and *Sinapis* *alba* L., ‘Asta’	Arbuscular mycorrhizal fungi (AMF) colonization, and *Fragaria x ananassa* Duch. var. ‘*Marmolada*’ strawberry yield	91 g of each biofumigant plant per kg of soil were added to AMF inoculated or not inoculated soil, corresponding to 78.9 mg GSL per kg of soil to the *B. juncea* treatment, 75.2 mg GSL per kg of soil to the *E. sativa* treatment and 28.6 mg GSL per kg of soil to the *S. alba* treatment. The soil treatments with biofumigant plants revealed moderate inhibitory effects on strawberry plant AMF colonization, whereas they increased the plant growth and fruit production, especially for the *B. juncea* and *S. alba* treatments. Effects of solarization were also investigated.	[168]
GSLs from *Brassica juncea*, *Raphanus sativus*, and *Sinapis alba*	*Verticillium dahlia* in vitro and in soils	Commercial standards of methyl ITC, propenyl AITC, 4-(methylsulfinyl)but-3-enyl4- methyl s u l f i n yl-3-butenyl ITC, benzyl ITC and 2-phenylethyl ITC were tested in vitro against V. dahliain sand at ITC concentrations of 1, 5, 25, 125, 625 nmol g^−1^ sand. Furthermore, the effect of propenyl ITC with a dose of 150 nmol g^−1^ on *V. dahliae* in natural infested soil samples from 22 sites with different crop rotation history and infestation levels was tested. All ITCs tested suppressed microsclerotia of *V. dahlia* in vitro in sterile sand, and the ITCs containing an aromatic moiety were considerably more toxic than the aliphatic ITCs. In natural soils the ITC toxicities seem negatively correlated to organic carbon content in the soils. In experiments with biomass incorporation in soil, *B. juncea* reduced the infection significatively (69-80% efficacy), while *S. alba* and *R. sativus* gave mortalities between 9-37%. Overall, the study demonstrates that brassicaceous green manures are hardly able to release ITCs at levels necessary for an adequate suppression of *V. dahlia* microsclerotia in natural soils and because organic matter can reduce the availability of ITCs and their effect. The authors conclude that more promising is the incorporation of high GSL-containing seed meal formulations, which should generate more effective ITC concentrations.	[169]
GSL-derived AITC released from *B. carinata* DSM	*Botrytis cinerea*, in vitro and in vivo with strawberries as plant host.	In in vitro trial AITC had a fungistatic effect against the pathogen. In in vitro trials two varieties of organic grown strawberries, infected with *B. cinerea* were exposed for 4 h in an atmosphere enriched either with synthetic AITC or ITC derived from DSM (0.1 mg L^−1^). The AITC treatment (pure or GSL-derived ITC) reduced the decay caused by the pathogen significantly different from the untreated fruit. Residue analysis performed on fruit at the end of storage showed values lower than 1 mg kg^−1^. Total phenolic content and antioxidant capacity estimated in treated and untreated strawberries showed no significant difference between control and AITC treated fruit.	[170]
**Glucosinolate/isothiocyanate**	**Target: Pseudofungi**	**Effect**	**References**
GSLs from DSMs of *Brassica napus*, *Brassica carinata* and *Brassica juncea* genotypes	*Phytophthora cinnamomi*, in vitro and *in planta* on *Lupinus luteus*	DSMs with high levels of allyl GSL inhibited mycelial growth and effectively inhibited the viability of chlamydospores in treated soils. Roots symptoms were less when plants grew in soils biofumigated with *B. carinata* and *B. juncea* DSMs with highest allyl GSL contents in comparison with plants in control soils. In particular *B. juncea* DSM (3 ÷ 30 µmol allyl GSL per gram of soil) had the largest effect on decreasing root necrosis by *P. cinnamomi* in *Lupinus*.	[171]
GSL from *Brassica carinata* pellets (Biofence)	*Phytophthora nicotianae* in vitro, in vivo with pepper plant as host.	Sensitivity of the vegetative structures of *P. nicotianae* to *Brassica carinata* pellets (Biofence) was evaluated in vitro at different doses and temperatures. The effectiveness of the pellets varied depending on the dose. The highest dose of pellets tested (24 mg) was fungitoxic to mycelium regardless of temperature for all the isolates. Moreover, biofumigation was effective in suppressing chlamydospores germination when the pellets were incorporated into the soil (1.5 and 3 g L−1 of soil) under different temperature regimes. In bioassays with pepper plants, both rates of *B. carinata* pellets (1.5 and 3 g L−1 of soil) reduced populations of *P. nicotianae* totally controlled the disease after a 4-week biofumigation treatment.	[172]
GSLs from above-ground parts of *B. napus*, *B. carinata* and *B. juncea* genotypes at different phenological stages	*Phytophthora cinnamomi*, in vitro and *in planta* on *Lupinus luteus*	Genotypes of Brassica with high levels of allyl GSL inhibited mycelial growth, decreased sporangial production, and effectively inhibited the viability of chlamydospores in soil, but only *B. carinata* (10 g/75 mL soil) decreased disease symptoms in *L. luteus* roots.	[173]
GSL from *B. carinata* pellets (Biofence)	*Phytophthora cinnamomi* in vitro and in vivo on *Quercus cerris*	Maximum inhibition of vegetative or reproductive structure in vitro occurred at 15 °C and decreased as temperature increased. In vivo assays confirmed efficacy of pellets (3 g L^−1^) in reducing the pathogen, but a total inhibition was not reached even if at high doses in comparison to the maximum dose tested in vitro assays (0.4 g L^−1^).	[174]
**Glucosinolate/isothiocyanate**	**Target: Insects, acari, and other arthropoda**	**Effect**	**References**
GSL from *Brassica juncea* granulated seed meal (Kosmalski Herbs & Spices)	*Melolontha melolontha* grubs	In dose–response experiments the mortality of the grubs at each instar was significantly dependent on the GSL concentration applied with the granulate. The mortality reached 100% in the smallest grubs at 320 µmol L^−1^, whereas at the same GSL concentration 95% of the bigger grubs (4.5÷7 mm) died. In field tests the mortality was 67.4%.	[175]
GSL derived from bio-based experimental formulations con-taining either *B. carinata* oil 1.5% and *B. carinata* DSM 3 g L^−1^ (270 µmol GSL L^−1^), or 2% *B. carinata* oil and *B. carinata* DSM 4.5 g L^−1^ (405 µmol GSL L^−1^).	red spider mite *Tetranychus urticae* on eggplant in open field trials.	The 2-year results indicated that the application of both formulations have a clear effect in containing mites, statistically different from the untreated control. Moreover, the ability of pest control of the formulation with the higher concentrations of oil and DSM was not different from the commercial chemical acaricide (fenazaquin),	[176]
GSL from leaf material of purple sprouting broccoli ‘Santee’, Savoy cabbage ‘Wintessa’, and the wild *B. oleracea* accession Winspit	*Folsomia candida* (springtail), *Eisenia andrei* (earthworm) and the soil bacterial community.	Biofumigation experiments were performed using the springtail *Folsomia candida* and the earthworm *Eisenia andrei*, each representing a functional soil invertebrate group with important effects on soil processes. Biofumigation was performed using freeze-dried leaves of the three different *B. oleracea* genotypes: One percent of freeze-dried leaf material relative to total soil (that is about 200 µmol kg^−1^ soil in GSL for Winspit accession; 125 µmol kg^−1^ soil in GSL for Santee accession, and 10 µmol kg^−1^ soil in GSL for Wintessa accession) was used for biofumigation. After 28 days, Winspit (but-3-enyl GSL as dominant GSL) was the genotype displaying highest toxicity to soil invertebrates. Earthworm survival was not affected by the *B. oleracea* plant material, and overall, the bacterial community was quite resilient to biofumigation.	[177]
GSLs derived by chopped fresh plants from *Brassica juncea*, sel. ISCI 99 and biofumigant meals derived from defatted seeds of *Brassica carinata* sel. ISCI 7	Wireworm populations (*Agriotes* *brevis* Candeze, *Agriotes sordidus* Illiger, and *Agriotes ustulatus* Schäller) was evaluated under both pot assays (on maize and lettuce) and field conditions on maize and potato.	In pot assays a clear rate effect was demonstrated, with sufficient seed meal to supply approximately 160 moles of GSL L^−1^ of soil resulting in significant wireworm mortality. At field level the protection of maize and potato crops comparable to that provided by Regent^®^.	[142]

**Table 3:** Biotechnological approaches to enhance glucosinolate (GSL) content.

Brassicaceae species	Biotechnological approach to enhance GSL content	Results	References
*Brassica oleracea var. Italica*	Introgression of three QTLs from *B. villosa* to commercial variety via classical breeding via MAS	Broccoli variety, Beneforté, with high level of ITC	[186]
*Brassica oleracea* var*. Italica* (broccoli) *x Brassica oleracea* var. *botrytis* (cauliflower) *Brassica oleracea var. Italica x Brassica oleracea var. Lacinato* *Brassica oleracea var.* *Lacinato* (collard) *x Brassica oleracea var.* *botrytis*	Classical breeding via MAS	RILs with high content of of 4-(methylsulfinyl)butyl and low (*R*)-2-hydroxybut-3-enyl GSL	[187]
*Brassica napus*	iRNA to target the GSL-ALK gene	*B. napus* iRNA lines with high content of of 4-(methylsulfinyl)butyl and low (*R*)-2-hydroxybut-3-enyl GSL	[188]
*A. thaliana*	Overexpression of *CYP79D2 and CYP79A1/A2* genes	p35S: *CYP79D2* plants with enhanced isopropyl and methylpropyl GSLs and resistant to *Erwinia carotovora*p35S: *CYP79A1/A2* plants were less susceptible to *P. syringe* but more susceptible to *A. brassiciola*	[190]
*A. thaliana*	Overexpression of *AOP2 gene*	Upregulation of aliphatic alkenyl GSL biosynthetic pathway	[192]
*A. thaliana*	Overexpression of *CYP79F1 and CYP79F2* genes	Hairy roots overexpressing the transgenes showed an increased aliphatic GSL rate compared to wild type roots but lower than leaf extract	[198]
*B. rapa x B. oleracea*	Conventional breeding via MAS	Cabbage lines with increased GSL content	[200]
*B. oleracea*	Kncock-out of *MYB28* gene via CRISPR/Cas9 mediated editing	downregulation of aliphatic GSL biosynthetic genes and reduction in methionine-derived GSL content in *myb28* mutant broccoli plants	[201]

## Data Availability

Data sharing is not applicable to this article. No new data were created or analyzed in this study.

## Abbreviations

AITC	allyl isotiocyanate
CA	3,4-Dihydroxyphenyl)prop-2-enoic acid
CQA	5-O-caffeoylquinic acid
CNV	copy number variation
CPB	Colorado Potato Beetle
DSMs	defatted seed meals
DW	dry weight
FW	fresh weight
GHPs	GSL hydrolysis products
GSLs	glucosinolates
HPLC	High Performance Liquid Chromatography
HPTLC	High Performance Thin Layer Chromatography
IPM	integrated pest management
ITCs	isotiocyanates
MAS	marker assisted selection
PPW	Potato Peel Waste
PTM	Potato Tuber Moth
QTL	quantitative trait locus
RIL	recombinant inbred line
SGAs	Steroidal glycoalkaloids
SA	steroidal alkaloid
SNPs	single nucleotide polymorphisms
UAE	Ultrasound-assisted extraction

## References

[1] Ishangulyyev R., Kim S., Lee S.H. (2019). Understanding Food Loss and Waste-Why Are We Losing and Wasting Food?. Foods.

[2] Köhler H.R., Triebskorn R. (2013). Wildlife Ecotoxicology of Pesticides: Can We Track Effects to the Population Level and Beyond?. Science.

[3] Keikotlhaile B.M., Spanoghe P., Steurbaut W. (2010). Effects of food processing on pesticide residues in fruits and vegetables: A meta-analysis approach. Food Chem. Toxicol..

[4] Yang L.N., He M.H., Ouyang H.B., Zhu W., Pan Z.C., Sui Q.J., Shang L.P., Zhan J. (2019). Cross-resistance of the pathogenic fungus *Alternaria alternata* to fungicides with different modes of action. BMC Microbiol..

[5] Directive 2009/128/EC of the European Parliament and of the Council of 21 October 2009, 2009. Establishing a Framework for Community Action to Achieve the Sustainable Use of Pesticides. Off. J. Eur. Union. https://www.europarl.europa.eu/RegData/etudes/STUD/2018/627113/EPRS_STU(2018)627113_EN.pdf.

[6] Poveda J., Eugui D., Velasco P. (2020). Natural control of plant pathogens through glucosinolates: An effective strategy against fungi and oomycetes. Phytochem. Rev..

[7] El-Mougy N.S., Abdel-Kader M.M. (2007). Antifungal effect of powdered spices and their extracts on growth and activity of some fungi in relation to damping-off disease control. J. Plant. Prot. Res..

[8] Abdel-Monaim M.F., Abo-Elyousr K.A.M., Morsy K.M. (2011). Effectiveness of plant extracts on suppression of damping-off and wilt diseases of lupine (Lupinus termis Forsik). Crop. Prot..

[9] Ahmed S.S., Abd El-Aziz G.H., Abou-Zeid M.A., Fahmy A.H. (2019). Environmental impact of the use of some eco-friendly natural fungicides to resist rust disease in wheat. CATRINA.

[10] Draz I.S., Elkhwaga A.A., Elzaawely A.A., El-Zahaby H.M., Anter Ismai A.W. (2019). Application of plant extracts as inducers to challenge leaf rust of wheat. Egypt J. Biol Pest. Control..

[11] Geetha H.M., Shetty H.S. (2012). Induction of resistance in pearl millet against mildew disease caused by Sclerospora graminicola using benzothiadiazole, calcium chloride and hydrogen peroxide—A comparative evaluation. Crop. Protection.

[12] Hassan M.E.M., Abd El-Rahman S.S., El-Abbasi I.H., Mikhail M.S. (2007). Change in peroxidase activity due to resistance induced against faba bean chocolate spot disease. Egypt J. Phytopathol..

[13] Radwan D.E., Lu G., Fayez K.A., Mahmoud S.Y. (2008). Protective action of salicylic acid against been yellow mosaic virus infection in *Vicia faba* leaves. J. Plant. Physiol..

[14] Benkeblia N. (2020). Potato Glycoalkaloids: Occurrence, biological activities and extraction for biovalorisation—A review. Int. J. Food Sci. Technol..

[15] FAO International Year of Potato. http://www.fao.org/potato-2008/en/potato/cultivation.html.

[16] Akyol H., Riciputi Y., Capanoglu E., Caboni M.F., VerardoInt V. (2016). Phenolic Compounds in the Potato and Its Byproducts: An Overview. Int. J. Mol. Sci..

[17] Sepelev I., Galoburda R. (2015). Industrial potato peel waste application in food production: A Review. Res. Rural Dev. Int. Sci. Conf. Proc. (Latvia).

[18] Samotyja U. (2019). Potato Peel as a Sustainable Resource of Natural Antioxidants for the Food Industry. Potato Res..

[19] Di W. (2016). Recycle Technology for Potato Peel Waste Processing: A Review. Procedia Env. Sci..

[20] Arapoglou D., Varzakas T., Vlyssides A., Israilides C. (2010). Ethanol production from potato peel waste (PPW). Waste Manage..

[21] Calcio Gaudino E., Colletti A., Grillo G., Tabasso S., Cravotto G. (2020). Emerging Processing Technologies for the Recovery of Valuable Bioactive Compounds from Potato Peels. Foods.

[22] Venturi F., Bartolini S., Sanmartin C., Orlando M., Taglieri I., Macaluso M., Lucchesini M., Trivellini A., Zinnai A., Mensuali A. (2019). Potato Peels as a Source of Novel Green Extracts Suitable as Antioxidant Additives for Fresh-Cut Fruits. Appl. Sci..

[23] Lazzeri L., Riva G., D’Avino L., Pedretti E.F. (2015). Short introduction to the VALSO and EXTRAVALORE project activities. Ind. Crops Prod..

[24] Fanigliulo R., Pochi D., Bondioli P., Grilli R., Fornaciari L., Folegatti L., Malaguti L., Matteo R., Ugolini L., Lazzeri L. (2021). Semi-refined Crambe abyssinica (Hochst. EX R.E.Fr.) oil as a biobased hydraulic fluid for agricultural applications. Biomass Conv. Bioref..

[25] D’Avino L., Dainelli R., Lazzeri L., Spugnoli P. (2015). The role of co-products in biorefinery sustainability: Energy allocation versus substitution method in rapeseed and carinata biodiesel chains. J. Clean. Prod..

[26] Maina S., Misinzo G., Bakari G., Kim H. (2020). Human, animal and plant health benefits of glucosinolates and strategies for enhanced bioactivity: A systematic review. Molecules.

[27] Chowa’nski S., Adamski Z., Marciniak P., Rosia’nski G., Büyükgüzel E., Büyükgüzel K., Falabella P., Scrano L., Ventrella E., Lelario F. (2016). Review of Bioinsecticidal Activity of *Solanaceae* Alkaloids. Toxin.

[28] Bennett R.N., Wallsgrove R.M. (1994). Secondary metabolites in plant defence mechanisms. New Phytologist.

[29] Pusztahelyi T., Holb U., Pocsi I. (2015). Secondary metabolites in fungal plant interaction. Front. Plant. Sci..

[30] De Sotillo D.R., Hadley M., Wolf-Hall C. (1998). Potato peel extract a nonmutagenic antioxidant with potential antimicrobial activity. J. Food Sci..

[31] Amanpour R., Abbasi-Maleki S., Neyriz-Naghadehi M., Asadi-Samani M. (2015). Antibacterial effects of *Solanum tuberosum* peel ethanol extract in vitro. J. HerbMed. Pharmacol..

[32] Friedman M., McDonald G.M., Filadelfi-Keszi M. (1997). Potato Glycoalkaloids: Chemistry, Analysis, Safety, and Plant Physiology. CRC Crit. Rev. Plant. Sci..

[33] Kozukue N., Yoon K.S., Byun G.I., Misoo S., Levin C.E., Friedman M. (2008). Distribution of glycoalkaloids in potato tubers of 59 accessions of two wild and five cultivated *Solanum* species. J. Agric. Food Chem..

[34] Manrique-Carpintero N.C., Tokuhisa J.G., Ginzberg I., Holliday J.A., Veilleux R.E. (2013). Sequence diversity in coding regions of candidate genes in the glycoalkaloid biosynthetic pathway of wild potato species. G3 Genes Genomes Genet..

[35] Mweetwa A.M., Hunter D., Poe R., Harich K.C., Ginzberg I., Veilleux R.E., Tokuhisa J.G. (2012). Steroidal glycoalkaloids in *Solanum chacoense*. Phytochemistry.

[36] Friedman M. (2006). Potato Glycoalkaloids and Metabolites: Roles in the Plant and in the Diet. J. Agric. Food Chem..

[37] Distl M., Wink M. (2009). Identification and Quantification of Steroidal Alkaloids from Wild Tuber-Bearing Solanum Species by HPLC and LC-ESI-MS. Potato Res..

[38] Itkin M., Heinig U., Tzfadia O., Bhide A.J., Shinde B., Cardenas P.D., Bocozeba S.E., Unger T., Malitsky S., Finkers R. (2013). Biosynthesis of antinutritional alkaloids in solanaceous crops is mediated by clustered genes. Science.

[39] Lafta A.M., Lorenzen J.H. (2000). Influence of High Temperature and Reduced Irradiance on Glycoalkaloid Levels in Potato Leaves. J. Am. Soc. Hort. Sci..

[40] Dale M.F.B., Griffiths D.W., Bain H., Todd D. (1993). Glycoalkaloid increase in *Solanum tuberosum* on exposure to light. Ann. Appl. Biol..

[41] Mekapogu M., Sohn H.-B., Kim S.-J., Lee Y.-Y., Park H.-M., Jin Y.-I., Hong S.-Y., Suh J.-T., Kweon K., Jeong J.-C. (2016). Effect of Light Quality on the Expression of Glycoalkaloid Biosynthetic Genes Contributing to Steroidal Glycoalkaloid Accumulation in Potato. Am. J. Potato Res..

[42] Choi D., Bostock R.M., Avdiushko S., Hildebrand O.F. (1994). Lipid-derived signals that discriminate wound- and pathogenresponsive isoprenoid pathways in plants: Methyl jasmonate and fungal elicitor arachidonic acid induce different 3-hydroxy3-methylglutaryl-coenzyme A reductase genes and antimicrobial isoprenoids in *Solanum tuberosum* L. Proc. Natl. Acad. Sci. USA.

[43] Zarins R., Kruma Z. (2017). Glycoalkaloids in potatoes: A review. Foodbalt.

[44] Friedman M., Roitman J.N., Kozukue N. (2003). Glycoalkaloid and calystegine contents of eight potato cultivars. J. Agric. Food Chem..

[45] Pacifico D., Musmeci S., Sanchez del Pulgar J., Onofri C., Parisi B., Sasso R., Mandolino G., Lombardi-Boccia G. (2019). Caffeic acid and α-chaconine influence the resistance of potato tuber to Phthorimaea operculella (Lepidoptera: Gelechiidae). Am. Potato J..

[46] Friedman M. (2005). Analysis of biologically active compounds in potatoes (*Solanum tuberosum* L.), tomatoes (*Lycopersicon esculentum* L.), and jimson weed (*Datura stramonium* L.) seeds. J. Chromatogr. A.

[47] Rytel E., Czopek A.T., Aniolowska M., Hamouz K. (2013). The influence of dehydrated potatoes processing on the glycoalkaloids content in coloured-fleshed potato. Food Chem..

[48] OECD Consensus document on compositional considerations for new varieties of potatoes: Key food and feed nutrients, anti-nutrients and toxicants. OECD Environmental Health and Safety Publications, Safety of Novel Foods and Feeds No. 4.

[49] BfR Opinion No 010/2018 of 23 April 2018. Table Potatoes Should Contain Low Levels of Glycoalkaloids (Solanine). http://www.bfr.bund.de/cm/349/table-potatoes-should-contain-low-levels-of-glycoalkaloids-solanine.pdf.

[50] Uluwaduge D.I. (2018). Glycoalkaloids, bitter tasting toxicants in potatoes: A review. Int. J. Food Sci. Nutr..

[51] Hellenäs K.E., Branzell C., Johnsson H., Slanina P. (1995). High levels of glycoalkaloids in the established Swedish potato variety Magnum Bonum. J. Sci. Food Agric..

[52] Knuthsen P., Jensen U., Schmidt B., Larsen I.K. (2009). Glycoalkaloids in potatoes: Content of glycoalkaloids in potatoes for consumption. J. Food Compos. Anal..

[53] Wu Z.G., Xu H.Y., Ma Q., Cao Y., Ma J.N., Ma C.M. (2012). Isolation, identification and quantification of unsaturated fatty acids, amides, phenolic compounds and glycoalkaloids from potato peel. Food Chem..

[54] Kremr D., Bajer T., Bajerová P., Surmová S., Ventura K. (2016). Unremitting problems with chlorogenic acid Nomenclature: A review. Quím. Nova.

[55] Sato Y., Itagaki S., Kurokawa T., Ogura J., Kobayashi M., Hirano T., Sugawara M., Iseki K. (2011). In vitro and in vivo antioxidant properties of chlorogenic acid and caffeic acid. Int. J. Pharm..

[56] Clifford M.N. (2000). Chlorogenic acids and other cinnamates—nature, occurrence, dietary burden, absorption and metabolism. J. Sci. Food Agric..

[57] Horbury D., Baker L.A., Quan W.D., Greenough S.E., Stavros W.G. (2016). Photodynamics of potent antioxidants: Ferulic and caffeic acids. Phys. Chem. Chem. Phys..

[58] Lucarini M., Pedulli G.F. (1994). Bond dissociation enthalpy of α-tocopherol and other phenolic antioxidants. J. Org. Chem..

[59] Le Bourvellec C., Renard C.M.G.C. (2012). Interactions between Polyphenols and Macromolecules: Quantification Methods and Mechanisms. Crit. Rev. Food Sci. Nutr..

[60] Santos-Buelga C., Scalbert A. (2000). Proanthocyanidins and tannin-like compounds—nature, occurrence, dietary intake and effects on nutrition and health. J. Sci. Food Agric..

[61] Grzesik M., Naparło K., Bartosz G., Sadowska-Bartosz I. (2018). Antioxidant properties of catechins: Comparison with other antioxidants. Food Chem.

[62] Veluri R., Weir T.L., Bais H.P., Stermitz F.R., Vivanco J.M. (2004). Phytotoxic and Antimicrobial Activities of Catechin Derivatives. J. Agric. Food Chem..

[63] Cipollini M.L., Levey D.J. (1997). Antifungal activity of Solanum fruit glycoalkaloids: Implications for frugivoryand seed dispersal. Ecology.

[64] Smith D.B., Roddick J.G., Jones J.L. (2001). Synergism between the potato glycoalkaloids alpha-chaconine and alpha-solanine in inhibition of snail feeding. Phytochemistry.

[65] Zitnak A., Filadelfi M.A. (1985). Estimation of taste thresholds of three potato glycoalkaloids. Can. Inst. Food Technol. J..

[66] Fewell A.M., Roddick J.G. (1993). Interactive antifungal activity of the glycoalkaloids α-solanine and α-chaconine. Phytochemistry.

[67] Fewell A.M., Roddick J.G., Weissenberg M.A. (1994). Interactions between the glycoalkaloids solasonine and solamargine in relation to inhibition of fungal growth. Phytochemistry.

[68] Friedman M., Huang V., Quiambao Q., Noritake S., Liu J., Kwon O., Chintalapati S., Young J., Levin C.E., Tam C. (2018). Potato Peels and Their Bioactive Glycoalkaloids and Phenolic Compounds Inhibit the Growth of Pathogenic Trichomonads. J. Agric. Food Chem..

[69] Marston A., Hostettmann K. (1984). Plant molluscicides. Phytochemistry.

[70] Nenaah G. (2011). Individual and synergistic toxicity of Solanaceous glycoalkaloids against two coleopteran stored-product insects. J. Pest. Sci..

[71] Gee J.M., Worley G.M., Jonhson I.T., Price K.R., Rutten A.A.J.J.J.L., Houben G.F., Penninks A.H. (1996). Effects of saponins and glycoalkaloids on the permeability and visibility of mammalian intestinal cells and on the integrity of tissue preparation in vitro. Toxicol. Vitr..

[72] Ruprich J., Rehurkova I., Boon P.E., Svensson K., Moussavian S., Van der Voet H., Bosgra S., Van Klaveren J.D., Busk L. (2009). Probabilistic modeling of exposure doses and implications for health risk characterization: Glycoalkaloids from potatoes. Food Chem Toxicol..

[73] Patel B., Schutte R., Sporns P., Doyle J., Jewel L., Fedorak R.N. (2002). Potato glycoalkaloids adversely affect intestinal permeability and aggravate inflammatory bowel disease. Inflamm. Bowel Dis..

[74] Blankemeyer J.Y., Stringer B.K., Rayburn J.R., Bantle J.A., Friedman M. (1992). Effect of potato glycoalkaloids, alpha-chaconine and alpha-solanine on membrane potential of frog embryos. J. Agric. Food Chem..

[75] Roddick J.G., Rijnenberg A.L., Weissenberg M. (1990). Membrane-disrupting properties of the steroidal glycoalkaloids solasonine and solamargine. Phytochemistry.

[76] Sarquis J.I., Coria N.A., Aguilar I., Rivera A. (2000). Glycoalkaloid content in *Solanum* species and hybrids from a breeding program for resistance to late blight (*Phytophthora infestans*). Am. J. Potato Res..

[77] Dahlin P., Müller M.C., Ekengren S., McKee L.S., Bulone V. (2017). The Impact of Steroidal Glycoalkaloids on the Physiology of Phytophthora infestans, the Causative Agent of Potato Late Blight. Mol. Plant. Microbe Interact..

[78] Weete J.W., Abril M., Blackwell M. (2010). Phylogenic distribution of fungal sterols. PLoS ONE.

[79] Roddick J.G., Rijnenberg A.L., Weissenberg M. (1992). Alterations to the permeability of liposome membranes by the solasodine-based glycoalkaloids solasonine and solamargine. Phytochemistry.

[80] Keukens E.A., de Vrije T., van den Boom C., de Waard P., Plasman H.H., Thiel F., Chupin V., Jongen W.M., de Kruijff B. (1995). Molecular basis of glycoalkaloid induced membrane disruption. Biochim. Biophys. Acta..

[81] Roddick J.G., Rijnenberg A.L., Osman S.F. (1988). Synergistic interaction between potato glycoalkaloids α-solanine and α-chaconine in relation to destabilization of cell membranes Ecological. Implic. J. Chem. Ecol..

[82] Sánchez-Maldonado A.F., Schieber A., Gänzle M.G. (2016). Antifungal activity of secondary plant metabolites from potatoes (*Solanum tuberosum* L.): Glycoalkaloids and phenolic acids show synergistic effects. J. Appl. Microbiol..

[83] Müller M.M., Kantola R., Kitunen V. (1994). Combining sterol and fatty acid profiles for the characterization of fungi. Mycol. Res..

[84] Mejanelle L., Lopez J.F., Gunde-Cimerman N., Grimalt J.O. (2000). Sterols of melanized fungi from hypersaline environments. Org. Geochem..

[85] Kesten C., Gámez-Arjona F.M., Menna A., Scholl S., Dora s., Huerta A.I., Huang H.Y., Tintor N., Kinoshita T., Rep M. (2019). Pathogen-induced pH changes regulate the growth-defense balance in plants. EMBO J..

[86] Ford J.E., McCance D.J., Drysdale R.B. (1977). The detoxification of α-tomatine by *Fusarium oxysporum* f. sp. Lycopersici Phytochemistry.

[87] Oda Y., Saito K., Ohara-Takada A., Mori M. (2002). Hydrolysis of the potato glycoalkaloids alpha-chaconine by filamentous fungi. J. Biosci. Bioeng..

[88] Hennessy R.C., Nielsen S.D., Greve-Poulsen M., Larsen L.B., Sørensen O.B., Stougaard P. (2020). Discovery of a Bacterial Gene Cluster for Deglycosylation of Toxic Potato Steroidal Glycoalkaloids α-Chaconine and α-Solanine. J. Agric. Food Chem..

[89] Andrews J.M. (2001). Determination of minimum inhibitory concentrations. J. Antimicrob. Chemother..

[90] Merkl R., Hrádková I., Filip V., Šmidrkal J. (2010). Antimicrobial and antioxidant properties of phenolic acids alkyl esters. Czech. J. Food Sci..

[91] Tsuchiya H. (1999). Effects of green tea catechins on membrane fluidity. Pharmacology.

[92] Deußer H., Guignard C., Hoffmann L., Evers D. (2012). Polyphenol and glycoalkaloid contents in potato cultivars grown in Luxembourg. Food Chem..

[93] Friedman M., Kozukue N., Kim H.J., Choi S.H., Mizuno M. (2017). Glycoalkaloid, phenolic, and flavonoid content and antioxidative activities of conventional nonorganic and organic potato peel powders from commercial gold, red, and Russet potatoes. J. Food Compos. Anal..

[94] Wang Y., Naber M.R., Crosby T.W. (2020). Effects of Wound-Healing Management on Potato Post-Harvest Storability. Agronomy.

[95] Janjai S., Bala B.K. (2012). Solar Drying Technology. Food Eng. Rev..

[96] Paolo D., Bianchi G., Morelli C.F., Speranza G., Campanelli G., Kidmose U., Lo Scalzo R. (2019). Impact of drying techniques, seasonal variation and organic growing on flavor compounds profiles in two Italian tomato varieties. Food Chem..

[97] Pandey A., Tripathi S. (2014). Concept of standardization, extraction and pre phytochemical screening strategies for herbal drug. J. Pharmacogn. Phytochem.

[98] Hossain M.B., Tiwari B.K., Gangopadhyay N., O’Donnell C.P., Brunton N.P., Rai D.K. (2014). Ultrasonic extraction of steroidal alkaloids from potato peel waste. Ultrason. Sonochem..

[99] Jie D., Wei X. (2018). Review on the recent progress of non-destructive detection technology for internal quality of watermelon. Comput. Electron. Agric..

[100] Sørensen K.K., Kirk H.G., Olsson K., Labouriau R., Christiansen J. (2008). A major QTL and an SSR marker associated with glycoalkaloid content in potato tubers from *Solanum tuberosum* × *S. sparsipilum* located on chromosome I. Theor. Appl. Genet..

[101] Krits P., Fogelman E., Ginzberg I. (2007). Potato steroidal glycoalkaloid levels and the expression of key isoprenoid metabolic genes. Planta.

[102] Khan M.S., Munir I., Khan I. (2013). The potential of unintended effects in potato glycoalkaloids. Afr. J. Biotechnol..

[103] Cui T., Bai J., Zhang J., Zhang J., Wang D. (2014). Transcriptional expression of seven key genes involved in steroidal glycoalkaloid biosynthesis in potato microtubers, N.Z.J. Crop. Hortic. Sci..

[104] Ginzberg I., Thippeswamy M., Fogelman E., Demirel U., Mweetwa A.M., Tokuhisa J., Veilleux R.E. (2012). Induction of potato steroidal glycoalkaloid biosynthetic pathway by overexpression of cDNA encoding primary metabolism HMG-CoA reductase and squalene synthase. Planta.

[105] Arnqvist L., Dutta P.C., Jonsson L., Sitbon F. (2003). Reduction of cholesterol and glycoalkaloid levels in transgenic potato plants by overexpression of a type 1 sterol methyltransferase cDNA. Plant. Physiol..

[106] Zook M.N., Kuc J.A. (1991). Induction of sesquiterpene cyclase and suppression of squalene synthetase activity in elicitor treated or fungal infected potato tuber tissue. Physiol. Mol. Plant. Pathol..

[107] Shi J., Gonzales R.A., Madan J., Bhattacharyya K. (1996). Identification and Characterization of an S-Adenosyl-l-methionine: D24-Sterol-C-methyltransferase cDNA from Soybean. J. Biol. Chem..

[108] Nurun N. (2011). Regulation of Sterol and Glycoalkaloid Biosynthesis in Potato (*Solanum tuberosum* L.)—Identification of Key Genes and Enzymatic Steps.

[109] Sawai S., Ohyama K., Yasumoto S., Seki H., Sakuma T., Yamamoto T., Takebayashi Y., Kojima M., Sakakibara H., Aoki T. (2014). Sterol side chain reductase 2 is a key enzyme in the biosynthesis of cholesterol, the common precursor of toxic steroidal glycoalkaloids in potato. Plant. Cell.

[110] Shepherd L., Hackett C., Alexander C., McNicol J., Sungurtas J., Stewart D., McCue K., Belknap W., Davies H. (2015). Modifying glycoalkaloid content in transgenic potato—Metabolome impacts. Food Chem..

[111] Manrique-Carpintero N.C., Tokuhisa J.G., Ginzberg I., Veilleux R.E. (2014). Allelic variation in genes contributing to glycoalkaloid biosynthesis in a diploid interspecific population of potato. Theor. Appl. Genet..

[112] Hardigan M., Laimbeer P., Newton L., Crisovan E., Hamilton J., Vaillancourt B., Wiegert-Rininger K., Wood J., Douches D., Farré E. (2017). Genome diversity of tuber-bearing *Solanum* uncovers complex evolutionary history and targets of domestication in the cultivated potato. Proc. Natl. Acad. Sci..

[113] Cárdenas P.D., Sonawane P., Pollier J., Bossche R.V., Dewangan V., Weithorn E., Tal L., Meir S., Rogachev I., Malitsky S. (2016). GAME9 regulates the biosynthesis of steroidal alkaloids and upstream isoprenoids in the plant mevalonate pathway. Nat. Commun..

[114] Manjulatha M., Hwang-Bae S., Yul-Ho K., Su-Jeong K., Kwang-Soo C., Oh-Keun K., Yong-Ik J., Su-Young H., Jeong-Hwan N., Jong-Taek S. (2014). Comparative Expression of Key Genes Involved in Steroidal Glycoalkaloid Biosynthesis in Tubers of Two Potato Cultivars, Atlantic and Haryoung. Plant. Breed. Biotechnol..

[115] Nahar N., Westerberg E., Arif U., Huchelmann A., Guasca A.O., Beste L., Dalman K., Dutta P.c., Jonsson L., Sitbon F. (2017). Transcript profiling of two potato cultivars during glycoalkaloid-inducing treatments shows differential expression of genes in sterol and glycoalkaloid metabolism. Sci. Rep..

[116] Mariot R.F., de Oliveira L.A., Voorhuijzen M.M., Staats M., Hutten R.C.B., van Dijk J.P., Kok E.J., Frazzon J. (2016). Characterization and Transcriptional Profile of Genes Involved in Glycoalkaloid Biosynthesis in New Varieties of *Solanum tuberosum* L. J. Agric. Food Chem..

[117] Sanford L.L., Deahl K.L., Sinden S.L., Ladd T.L. (1992). Glycoalkaloid contents in tubers from Solanum tuberosum populations selected for potato leafhopper resistance. Am. Potato J..

[118] Zhang W., Zuo C., Chen Z., Kang Y., Qin S. (2019). RNA Sequencing Reveals That Both Abiotic and Biotic Stress-Responsive Genes Are Induced during Expression of Steroidal Glycoalkaloid in Potato Tuber Subjected to Light Exposure. Genes.

[119] Bengtsson T., Weighill D., Proux-Wéra E., Levander F., Resjö S., Burra D.D., Moushib L.I., Hedley P.E., Liljeroth E., Jacobson D. (2014). Proteomics and transcriptomics of the BABA-induced resistance response in potato using a novel functional annotation approach. BMC Genom..

[120] Paudel J.R., Davidson C., Song J., Maxim I., Aharoni A., Tai H.H. (2017). Pathogen and pest responses are altered due to RNAi-mediated knockdown of Lycoalkaloid Metabolism4 in *Solanum tuberosum*. Mol. Plant. Microbe Interact..

[121] Blažević I., Montaut S., Burčul F., Olsen C.E., Burow M., Rollin P., Agerbirk N. (2020). Glucosinolate structural diversity, identification, chemical synthesis and metabolism in plants. Phytochemistry.

[122] Halkier B.A., Gershenzon J. (2006). Biology and biochemistry of glucosinolates. Annu. Rev. Plant. Biol..

[123] Buxdorf K., Yaffe H., Barda O., Levy M. (2013). The effects of glucosinolates and their breakdown products on necrotrophic fungi. PLoS ONE.

[124] Galletti S., Sala E., Leoni O., Cinti S., Cerato C. (2008). *Aspergillus flavus* transformation of glucosinolates to nitriles by an arylsulfatase and a β-thio-glucosidase. Soil Biol. Biochem..

[125] Züst T., Strickler S.R., Powell A.F., Mabry M.E., An H., Mirzaei M., York T., Holland C.K., Kumar P., Erb M. (2020). Independent evolution of ancestral and novel defenses in a genus of toxic plants (*Erysimum*, Brassicaceae). eLife.

[126] Barco B., Clay N.K. (2019). Evolution of glucosinolate diversity via whole-genome duplications, gene rearrangements, and substrate promiscuity. Annu. Rev. Plant. Biol..

[127] Chhajed S., Misra B.B., Tello N., Chen S. (2019). Chemodiversity of the glucosinolate-myrosinase system at the single cell type resolution. Front. Plant. Sci..

[128] Zhang K.X., Hao H.Y., Li J. (2017). Recent research advances in the mustard oil glycoside-black mustard enzyme defense system. J. Plant. Physiol..

[129] Jang M., Hong E., Kim G.H. (2010). Evaluation of antibacterial activity of 3-butenyl, 4-pentenyl, 2-phenylethyl, and benzyl isothiocyanate in *Brassica* vegetables. J. Food Sci..

[130] Melrose J. (2019). The Glucosinolates: A sulphur glucoside family of mustard anti-tumour and antimicrobial phytochemicals of potential therapeutic application. Biomedicines.

[131] Pane C., Villecco D., Roscigno G., De Falco E., Zaccardelli M. (2013). Screening of plant-derived antifungal substances useful for the control of seedborne pathogens. Arch. Phytopathology Plant. Protect..

[132] Tierens K.F.J., Thomma B.P., Brouwer M., Schmidt J., Kistner K., Porzel A., Broekaert W.F. (2001). Study of the role of antimicrobial glucosinolate-derived isothiocyanates in resistance of *Arabidopsis* to microbial pathogens. Plant. Physiol..

[133] Zhang M., Li Y., Bi Y., Wang T., Dong Y., Yang Q., Zhang T. (2020). 2-Phenylethyl isothiocyanate exerts antifungal activity against Alternaria alternata by affecting membrane integrity and mycotoxin production. Toxins.

[134] Nazareth T.M., Corrêa J.A.F., Pinto A.C.S.M., Palma J.B., Meca G., Bordin K., Luciano F.B. (2018). Evaluation of gaseous allyl isothiocyanate against the growth of mycotoxigenic fungi and mycotoxin production in corn stored for 6 months. J. Sci. Food Agric..

[135] Nazareth T.D.M., Alonso-Garrido M., Stanciu O., Mañes J., Manyes L., Meca G. (2020). Effect of allyl isothiocyanate on transcriptional profile, aflatoxin synthesis, and *Aspergillus flavus* growth. Int. Food Res. J..

[136] Tracz B.L., Bordin K., Nazareth T.D.M., Costa L.B., Macedo R.E.F.D., Meca G., Luciano F.B. (2017). Assessment of allyl isothiocyanate as a fumigant to avoid mycotoxin production during corn storage. LWT.

[137] Azaiez I., Meca G., Manyes L., Luciano F.B., Fernández-Franzón M. (2013). Study of the chemical reduction of the fumonisins toxicity using allyl, benzyl and phenyl isothiocyanate in model solution and in food products. Toxicon.

[138] Azaiez I., Meca G., Manyes L., Fernández-Franzón M. (2013). Antifungal activity of gaseous allyl, benzyl and phenyl isothiocyanate in vitro and their use for fumonisins reduction in bread. Food Control..

[139] Quiles J.M., Manyes L., Luciano F.B., Mañes J., Meca G. (2015). Effect of the oriental and yellow mustard flours as natural preservative against aflatoxins B1, B2, G1 and G2 production in wheat tortillas. Int. J. Food Sci. Technol..

[140] Lazzeri L., Curto G., Leoni O., Dallavalle E. (2004). Effects of glucosinolates and their enzymatic hydrolysis products via myrosinase on the root-knot nematode *Meloidogyne incognita* (Kofoid et White) Chitw. J. Agric. Food Chem..

[141] Bhushan S., Gupta S., Sohal S.K., Arora S. (2016). Assessment of insecticidal action of 3-Isothiocyanato-1-propene on the growth and development of *Spodoptera litura* (Fab.) (Lepidoptera: Noctuidae). J. Entomol. Zool. Stud..

[142] Furlan L., Bonetto C., Finotto A., Lazzeri L., Malaguti L., Patalano G., Parker W. (2010). The efficacy of biofumigant meals and plants to control wireworm populations. Ind. Crops Prod..

[143] Wu H., Zhang G.A., Zeng S., Lin K.C. (2009). Extraction of allyl isothiocyanate from horseradish (*Armoracia rusticana*) and its fumigant insecticidal activity on four stored-product pests of paddy. Pest. Manag. Sci..

[144] Freitas R.C.P., Faroni L.R.D., Haddi K., Jumbo V.L.O., Oliveira E.E. (2016). Allyl isothiocyanate actions on populations of *Sitophilus zeamais* resistant to phosphine: Toxicity, emergence inhibition and repellency. J. Stored Prod. Res..

[145] Messiha N.K., Ahmed S.A., El-Rokiek K.G., Dawood M.G., El-Masry R.R. (2013). The physiological influence of allelochemicals in two *Brassicaceae* plant seeds on the growth and propagative capacity of *Cyprus rotundus* and *Zea mays* L. World Appl. Sci. J..

[146] Smith J.D., Woldemariam M.G., Mescher M.C., Jander G., De Moraes C.M. (2016). Glucosinolates from host plants influence growth of the parasitic plant *Cuscuta gronovii* and its susceptibility to aphid feeding. Plant. Physiol..

[147] Lazzeri L., Curto G., Dallavalle E., D’Avino L., Malaguti L., Santi R., Patalano G. (2009). Nematicidal efficacy of biofumigation by defatted brassicaceae meal for control of Meloidogyne incognita (Kofoid et White) Chitw. on a full field zucchini crop. Int. J. Agric. Sustain..

[148] Ntalli N., Caboni P. (2017). A review of isothiocyanates biofumigation activity on plant parasitic nematodes. Phytochem. Rev..

[149] Curto G., Dallavalle E., Matteo R., Lazzeri L. (2016). Biofumigant effect of new defatted seed meals against the southern root-knot nematode, Meloidogyne incognita. Ann. Appl. Biol..

[150] Meyer S.L.F., Zasada I.A., Orisajo S.B., Morra M.J. (2011). Mustard seed meal mixtures: Management of Meloidogyne incognita on pepper and potential phytotoxicity. J. Nematol..

[151] Laquale S., Candido V., Avato P., Argentieri M.P., D’addabbo T. (2015). Essential oils as soil biofumigants for the control of the root-knot nematode Meloidogyne incognita on tomato. Ann. Appl. Biol..

[152] (2020). Ozone Secretariat. https://ozone.unep.org/sites/default/files/Handbooks/MP-Handbook-2020-English.pdf.

[153] Lazzeri L., Baruzzi G., Malaguti L., Antoniacci L. (2003). Replacing methyl bromide in annual strawberry production with glucosinolate-containing green manure crops. Pest. Manag. Sci..

[154] Curto G., Dallavalle E., Lazzeri L. (2005). Life cycle duration of *Meloidogyne incognita* and host status of Brassicaceae and Capparaceae selected for glucosinate content. Nematology.

[155] Lazzeri L., Leoni O., Manici L.M., Palmieri S., Patalano G. (2010). Use of Seed Flour as Soil Pesticide. Patent US.

[156] Lazzeri L., D’Avino L., Ugolini L., De Nicola G.R., Cinti S., Malaguti L., Bagatta M., Patalano G., Leoni O., Lazzeri L. Bio-based products from Brassica carinata A. Braun oils and defatted meals by a second generation biorefinery approach. Proceedings of the 19th European Biomass Conference.

[157] Ntalli N., Adamski Z., Doula M., Monokrousos N. (2020). Nematicidal amendments and soil remediation. Plants.

[158] Paudel B.R., Carpenter-Boggs L., Higgins S. (2016). Influence of brassicaceous soil amendments on potentially beneficial and pathogenic soil microorganisms and seedling growth in Douglas-fir nurseries. Appl. Soil Ecol..

[159] Lazzeri L., Malaguti L., Cinti S., Ugolini L., De Nicola G.R., Bagatta M., Casadei N., D’avino L., Matteo R., Patalano G. (2013). The *Brassicaceae* biofumigation system for plant cultivation and defence. An Italian twenty-year experience of study and application. Acta Hortic..

[160] Argento S., Melilli M.G., Branca F. (2019). Enhancing Greenhouse Tomato-Crop Productivity by Using *Brassica macrocarpa* Guss. Leaves for controlling root-knot nematodes. Agronomy.

[161] Ngala B.M., Haydock P.P., Woods S., Back M.A. (2015). Biofumigation with *Brassica juncea*, *Raphanus sativus* and *Eruca sativa* for the management of field populations of the potato cyst nematode *Globodera pallida*. Pest. Manag. Sci..

[162] Mocali S., Landi S., Curto G., Dallavalle E., Infantino A., Colzi C., D’Errico G., Roversi P.F., D’Avino L., Lazzeri L. (2015). Resilience of soil microbial and nematode communities after biofumigant treatment with defatted seed meals. Ind. Crops Prod..

[163] Rudolph R.E., Sams C., Steiner R., Thomas S.H., Walker S., Uchanski M.E. (2015). Biofumigation Performance of Four *Brassica* Crops in a Green Chile Pepper (*Capsicum annuum*) Rotation System in Southern New Mexico. HortScience.

[164] Argento S., Raccuia S.A., Melilli M.G., Toscano V., Branca F. (2013). Brassicas and their glucosinolate content for the biological control of root-knot nematodes in protected cultivation. Acta Hortic..

[165] Vandicke J., De Visschere K., Deconinck S., Leenknecht D., Vermeir P., Audenaert K., Haesaert G. (2020). Uncovering the biofumigant capacity of allyl isothiocyanate from several *Brassicaceae* crops against *Fusarium* pathogens in maize. J. Sci. Food Agric..

[166] Piccinini E., Ferrari V., Campanelli G., Fusari F., Righetti L., Pagnotta E., Lazzeri L. (2015). Effect of two liquid formulations based on *Brassica carinata* co-products in containing powdery mildew on melon. Ind. Crops Prod..

[167] Handiseni M., Jo Y., Lee K., Zhou X. (2016). Screening brassicaceous plants as biofumigants for management of Rhizoctonia solani AG1-IA. Plant. Dis..

[168] Koron D., Sonjak S., Regvar M. (2014). Effects of non-chemical soil fumigant treatments on root colonisation with arbuscular mycorrhizal fungi and strawberry fruit production. Crop. Prot..

[169] Neubauer C., Heitmann B., Müller C. (2004). Biofumigation potential of Brassicaceae cultivars to *Verticillium dahliae*. Eur. J. Plant. Pathol..

[170] Ugolini L., Martini C., Lazzeri L., D’Avino L., Mari M. (2014). Control of postharvest grey mould (*Botrytis cinerea* Per.: Fr.) on strawberries by glucosinolate-derived allyl-isothiocyanate treatments. Postharvest Biol. Technol..

[171] Ríos P., González M., Obregón S., Carbonero M., Leal J., Fernández P., De Haro A., Sánchez M. (2017). Brassica-based seedmeal biofumigation to control *Phytophthora cinnamomi* in the Spanish “dehesa” oak trees. Phytopathol. Mediterr..

[172] Serrano- Pérez P., Palo C., Rodríguez -Molina M.D.C. (2017). Efficacy of *Brassica carinata* pellets to inhibit mycelial growth and chlamydospores germination of *Phytophthora nicotianae* at different temperature regimes. Sci. Hortic..

[173] Ríos P., Obregón S., De Haro A., Fernández -Rebollo P., Serrano M., Sánchez M. (2016). Effect of *Brassica* Biofumigant Amendments on Different Stages of the Life Cycle of *Phytophthora cinnamomi*. J. Phytopathol. (1986).

[174] Morales-Rodríguez C., Vettraino A.M., Vannini A. (2016). Efficacy of biofumigation with *Brassica carinata* commercial pellets (BioFence) to control vegetative and reproductive structures of *Phytophthora cinnamomi*. Plant. Dis..

[175] Sukovata L., Jaworski T., Kolk A. (2015). Efficacy of *Brassica juncea* granulated seed meal against *Melolontha* grubs. Ind. Crops Prod..

[176] Piccinini E., Ferrari V., Campanelli G., Fusari F., Righetti L., Matteo R., Lazzeri L. (2015). Effect of two bio-based liquid formulations from *Brassica carinata* in containing red spider mite (*Tetranychus urticae*) on eggplant. Ind. Crops Prod..

[177] Zuluaga D.L., Van Ommen Kloeke A.E.E., Verkerk R., Röling W.F.M., Ellers J., Roelofs D., Aarts M.G.M. (2015). Biofumigation using a wild *Brassica oleracea* accession with high glucosinolate content affects beneficial soil invertebrates. Plant. Soil.

[178] Kirkegaard J.A., Matthiessen J. (2004). Developing and refining the biofumigation concept. Agroindustria.

[179] Lu P., Gilardi G., Gullino M.L., Garibaldi A. (2010). Biofumigation with Brassica plants and its effect on the inoculum potential of *Fusarium* yellows of *Brassica* crops. Eur. J. Plant. Pathol..

[180] Regulation (EC) No 1907/2006 of the European Parliament and of the Council of 18 December 2006 concerning the Registration, Evaluation, Authorisation and Restriction of Chemicals (REACH). https://eur-lex.europa.eu/legal-content/en/ALL/?uri=CELEX%3A32006R1907.

[181] Matteo R., Back M.A., Reade J.P.H., Ugolini L., Pagnotta E., Lazzeri L. (2018). Effectiveness of defatted seed meals from Brassicaceae with or without crude glycerin against black grass (*Alopecurus myosuroides* Huds.). Ind. Crops Prod..

[182] Brown P.D., Morra M.J. (1997). Control of soil-borne plant pests using glucosinolate-containing plants. Adv. Agron..

[183] Schnug E., Haneklaus S. (2016). Glucosinolates—the agricultural story. In: Kopriva, S., ed. Glucosinolates. Adv. Bot. Res..

[184] Gupta S.K. (2016). Brassicas. Breeding Oilseed Crops for Sustainable Production.

[185] Faulkner K., Mithen R., Williamson G. (1998). Selective increase of the potential anticarcinogen 4-methylsulphinylbutyl glucosinolate in broccoli. Carcinogenesis.

[186] Mithen R., Faulkner K., Magrath R., Rose P., Williamson G., Marquez J. (2003). Development of isothiocyanate enriched broccoli, and its enhanced ability to induce phase 2 detoxification enzymes in mammalian cells. Theor. Appl. Genet..

[187] Li G., Riaz A., Goyal S., Abel S., Quiros C.F. (2001). Inheritance of Three Major Genes Involved in the Synthesis of Aliphatic Glucosinolates in *Brassica oleracea*. J. Am. Soc. Hort. Sci..

[188] Liu Z., Hirani A.H., McVetty P.B.E., Daayf F., Quiros C.F., Li G. (2012). Reducing progoitrin and enriching glucoraphanin in *Brassica napus* seeds through silencing of the GSL-ALK gene family. Plant. Mol. Biol..

[189] Petersen A., Wang C., Crocoll C., Halkier B.A. (2018). Biotechnological approaches in glucosinolate production. J. Integr. Plant. Biol..

[190] Brader G., Mikkelsen M.D., Halkier B.A., Tapio Palva E. (2006). Altering glucosinolate profiles modulates disease resistance in plants. Plant. J..

[191] Wentzell A.M., Rowe H.C., Hansen B.G., Ticconi C., Halkier B.A., Kliebenstein D.J. (2007). Linking metabolic QTL with network and cis-eQTLs controlling biosynthetic pathways. PLoS Genet..

[192] Neal C.S., Fredericks D.P., Griffiths C.A., Neale A.D. (2010). The characterisation of AOP2: A gene associated with the biosynthesis of aliphatic alkenyl glucosinolates in *Arabidopsis thaliana*. BMC Plant. Biol..

[193] Bell L., Chadwick M., Puranik M., Tudor R., Methven L., Kennedy S., Wagstaff C. (2020). The *Eruca sativa* Genome and Transcriptome: A Targeted Analysis of Sulfur Metabolism and Glucosinolate Biosynthesis Pre and Postharvest. Front. Plant. Sci..

[194] Falk K.L., Tokuhisa J.G., Gershenzon J. (2007). The effect of sulfur nutrition on plant glucosinolate content: Physiology and molecular mechanisms. Plant. Biol. (Stuttg).

[195] Mithen R., Ho E. (2018). Isothiocyanates for Human Health. Mol. Nutr Food Res..

[196] Traka M.H., Saha S., Huseby S., Kopriva S., Walley P.G., Barker G.C., Moore J., Mero G., van den Bosch F., Constant H. (2013). Genetic regulation of glucoraphanin accumulation in Benefortè broccoli. New Phytol..

[197] Ruffoni B., Pistelli L., Bertoli A., Pistelli L. (2010). Plant cell cultures: Bioreactors for industrial production. Adv. Exp. Med. Biol..

[198] Kastell A., Zrenner R., Schreiner M., Kroh L., Ulrichs C., Smetanska I., Mewis I. (2015). Metabolic engineering of aliphatic glucosinolates in hairy root cultures of *Arabidopsis thaliana*. Plant. Mol. Biol. Rep..

[199] Wielanek M., Urbanek H. (2006). Enhanced glucotropaeolin production in hairy root cultures of *Tropaeolum majus* L. by combining elicitation and precursor feeding. Plant. Cell Tiss. Organ. Cult..

[200] Hirani A.H., Li G., Zelmer C.D., McVetty P.B.E., Asif M., Goyal A., Goyal A. (2012). Molecular genetics of glucosinolate biosynthesis in Brassicas: Genetic manipulation and application aspects. Crop Plant.

[201] Neequaye M., Stavnstrup S., Lawrenson T., Hundleby P., Troncoso-Rey P., Saha S., Harwood W., Traka H.M., Mithen R., Østergaard L. (2020). CRISPR-Cas9-mediated editing of myb28 impairs glucoraphanin accumulation of *Brassica oleracea* in the field. bioRxiv Preprint.

[202] Gao M., Li G., Yang B., Qiu D., Farnham M., Quiros C. (2007). High-density *Brassica oleracea* linkage map: Identification of useful new linkages. Theor. Appl. Genet..

[203] Issa R.A. (2010). Identification of glucosinolate profile in *Brassica oleracea* for quantitative trait locus mapping. Ph.D. Thesis.

[204] Hirani A.H. (2011). QTL mapping, gene identification and genetic manipulation of glucosinolates in *Brassica rapa* L. Ph.D. Thesis.

[205] Lou P., Zhao J., He H., Hanhart C., Del Carpio D.P., Verkerk R., Custers J., Koornneef M., Bonnema G. (2008). Quantitative trait loci for glucosinolate accumulation in *Brassica rapa* leaves. New Phytol..

[206] Ramchiary N., Padmaja K.L., Sharma S., Gupta V., Sodhi Y.S., Mukhopadhyay A., Arumugam N., Pental D., Pradhan A.K. (2007). Mapping of yield influencing QTL in *Brassica juncea*: Implications for breeding of a major oilseed crop of dryland areas. Theor. Appl. Genet..

[207] Hirani A.H., Geng J., Zhang J., Zelmer C.D., McVetty P.B.E., Daayf F., Li G. (2016). Quantitative Trait Loci Mapping and Candidate Gene Identification for Seed Glucosinolates in *Brassica rapa* L. Crop. Sci..

[208] Zhang J., Wang H., Liu Z., Liang J., Wu J., Cheng F., Mei S., Wang X. (2018). A naturally occurring variation in the BrMAM-3 gene is associated with aliphatic glucosinolate accumulation in *Brassica rapa* leaves. Hortic. Res..

[209] Quijada P.A., Udall J.A., Lambert B., Osborn T.C. (2006). Quantitative trait analysis of seed yield and other complex traits in hybrid spring rapeseed (*Brassica napus* L.): Identification of genomic regions from winter germplasm. Theor. Appl. Genet..

[210] Hasan M., Friedt W., Pons-Kuhnemann J., Freitag N.M., Link K., Snowdon R.J. (2008). Association of gene-linked SSR markers to seed glucosinolate content in oilseed rape (*Brassica napus* ssp. *napus*). Theor. Appl. Genet..

[211] Lionneton E., Aubert G., Ochatt S., Merah O. (2004). Genetic analysis of agronomic and quality traits in mustard (*Brassica juncea*). Theor. Appl. Genet.

[212] Ripley V.L., Roslinsky V. (2005). Identification of an ISSR marker for 2-propenyl glucosinolate content in *Brassica juncea* L. and conversion to a SCAR marker. Mol. Breed..

[213] Howell P.M., Sharpe A.G., Lydiate D.J. (2003). Homoeologous loci control the accumulation of seed glucosinolates in oilseed rape (*Brassica napus*). Genome.

[214] Mahmood T., Ekuere U., Yeh F., Good A.G., Stringam G.R. (2003). Molecular mapping of seed aliphatic glucosinolates in *Brassica juncea*. Genome.

[215] Zou Z., Ishida M., Li F., Kakizaki T., Suzuki S., Kitashiba H., Nishio T. (2013). QTL analysis using SNP markers developed by next-generation sequencing for identification of candidate genes controlling 4-methylthio-3-butenyl glucosinolate contents in roots of radish, *Raphanus sativus* L. PLoS ONE.

[216] Javidfar F., Cheng B. (2013). Construction of a genetic linkage map and QTL analysis of erucic acid content and glucosinolate components in yellow mustard (*Sinapis alba* L.). BMC Plant. Biol..

[217] Cardellina J.H., Gulter H.G. (1988). Biologically natural products in the search for new agrochemicals. Biologically active natural products: Potential use in agriculture.

[218] Gulter H.G., Gulter H.G. (1998). Natural products and their potential in agriculture: A personal overview. Biologically active natural products: Potential use in agriculture.

[219] Emosairue S.O., Ukeh D.A. (1996). Field trial of neem products for the control of okra flea beetles (*Podagrica* spp) in South Eastern Nigeria. Afr. J. Plant. Prot..

[220] Tewari S.N., Nayak M. (1991). Activity of four-plant leaf extracts against three fungal pathogens of rice. Trop. Agric. (Trinidad).

[221] Al-Abed A.S., Quasem J.R., Abu-Blan H.A. (1993). Antifungal effects of some common wild plant species on certain plant pathogenic fungi. Dirasat (Pure Appl. Sci.).

[222] Amadioha A.C. (1998). Control of powdery mildew of pepper (*Capsicum annum L*) by leaf extracts of papaya (*Asimina triloba*). J. Herbs Spices Med. Plants.

[223] Amadioha A.C. (2000). Controlling rice blast in vitro and in vivo with extracts of *Azadirachta indica*. Crop. Prot..

[224] Amadioha A.C., Obi V.I. (1998). Fungitoxic activity of extracts from *Azadirachta indica* and *Xylopia aethiopica* on *Colletotrichum lindemuthianum* in Cowpea. J. Herbs Spices Med. Plants.

[225] Mason J.R., Mathew D.N. (1996). Evaluation of neem as a bird repellent chemical. Int. J. Pest Manag.

[226] Metcalf R.L., Metclaf R.A., Rhodes A.N. (1980). Cucurbitacins as Kairomones for diabroticide beetles. Proc. Natl. Acad. Sci. USA.

[227] Neilson J.K., Larsen L.M., Sorenson H.J. (1977). Cucurbitacins E and I in *Iberis amara*, feeding inhibitors for *Phyllotreta nemorum*. Phytochemistry.

[228] Singh D.C. Scope of medicinal and aromatic plants in pest management. Proceedings of the International Symposium, Allelopathy in sustainable Agriculture, Forestry and Environment.

[229] Fandohan P., Gbenou J.D., Gnonlonfin B., Hell K., Marasas F.O., Wingfield M.G. (2004). Effect of essential oils on the growth of Fusarium verticillioides and fumonisin contamination in corn. J. Agric. Food Chem..

[230] Osbourn A.E. (1999). Antimicrobial phytoprotectants and fungal pathogens: A commentary. Fungal Genet. Biol..

[231] Krishnamurthy K., Balconi C., Sherwood J.E., Giroux M. (2001). Increased tolerance to fungal diseases of rice plants transformed with puroindoline genes. Mol. Plant. Microbe Interact..

[232] Ferreira R.B., Monteiro S., Freitas R., Santos C.N., Chen Z., Batista L.M., Duarte J., Borges A., Teixeira A.R. (2007). The role of plant defence proteins in fungal pathogenesis. Mol. Plant. Pathol..

[233] Balconi C., Lanzanova C., Motto M., Martin R.J. (2010). Ribosome-inactivating proteins in cereals. Toxic Plant Proteins.

[234] Punja Z. (2001). Genetic engineering of plants to enhance resistance to fungal pathogens—A review of progress and future prospects. Can. J. Plant. Pathol..

[235] Tarchevsky I.A. (2001). Pathogen-Induced Plant Proteins (Review). Appl. Biochem. Microbiol..

[236] Keen N.T. (1999). Plant disease resistance: Progress in basic understanding and practical application. Adv. Bot Res..

[237] Balconi C., Stevanato P., Motto M., Biancardi E., Ashraf M., Ozturk M., Ahmad M.S.A., Aksoy A. (2012). Breeding for biotic stress resistance/tolerance in plants In Crop. Production for Agricultural Improvement.

[238] Mesterházy A., Oláh J., Popp J. (2020). Losses in the Grain Supply Chain: Causes and Solutions. Sustainability.

[239] Munkvold G.P. (2003). Cultural and genetic approaches to managing mycotoxins in maize. Annu. Rev. Phytopathol..

[240] Balconi C., Berardo N., Locatelli S., Lanzanova C., Torri A., Redaelli R. (2014). Evaluation of ear rot (*Fusarium verticillioides*) resistance and fumonisin accumulation in Italian maize inbred lines. Phytopathol. Mediterr..

[241] Balconi C., Motto M., Mazzinelli G., Berardo N. (2010). Ear secondary traits related to aflatoxin accumulation in commercial maize hybrids under artificial field inoculation. World Mycotoxin J..

[242] Castegnaro M., McGregor D. (1998). Carcinogenic risk assessment of mycotoxins. Rev. Med. Vet..

[243] CAST (2003). Mycotoxins: Risks in Plant, Animal, and Human Systems.

[244] Lanzanova C., Torri A., Motto M., Balconi C. (2011). Characterization and interaction between b-32 maize ribosome-inactivating protein and fungal pathogens development in vivo and in vitro. Maydica.

[245] Balazs E., Schepers J.S. (2007). The mycotoxin threat to world safety. Int. J. Food Microbiol..

[246] Berardo N., Lanzanova C., Locatelli S., Laganà P., Verderio A., Motto M. (2011). Levels of total fumonisins in maize samples from Italy during 2006–2008. Food Addit. Contam. Part. B.

[247] European Commission Regulation (EC) No 1881/2006 of 19 December 2006 Setting Maximum Levels for Certain Contaminants in Foodstuffs. https://eur-lex.europa.eu/legal-content/EN/ALL/?uri=CELEX%3A32006R1881.

[248] European Commission Regulation (EC) No 1126/2007 of 28 September 2007 Amending Regulation (EC) No 1881/2006 Setting Maximum Levels for Certain Contaminants in Foodstuffs as Regards Fusarium Toxins in Maize and Maize Products. https://eur-lex.europa.eu/legal-content/EN/ALL/?uri=CELEX%3A32007R1126.

[249] Hartings H., Lanzanova C., Lazzaroni N., Balconi C. (2016). How maize trackles *Diabrotica v.* virgifera attack. Maydica.

[250] Herrera-Foessel S.A., Singh R.P., Huerta-Espino J., Crossa J., Yuen J., Djurle A. (2006). Effect of leaf rust on grain yield and yield traits of durum wheats with race-specific and slow-rusting resistance to leaf rust. Plant. Dis..

[251] Savary S., Willocquet L., Pethybridge S., Esker P., McRoberts N., Nelson A. (2019). The global burden of pathogens and pests on major food crops. Nat. Ecol. Evol..

